# Noise-Robust Bearing Fault Diagnosis via Soft-VMD and Hierarchical Feature Fusion

**DOI:** 10.3390/s26216841

**Published:** 2026-10-29

**Authors:** Yichen Liu, Luping Wang

**Affiliations:** College of Automation, Shenyang Aerospace University, 37 Daoyi South Street, Shenbei New District, Shenyang 110136, China; liuyichen2@stu.sau.edu.cn

**Keywords:** bearing fault diagnosis, variational mode decomposition, modal soft-suppression reconstruction, refined composite multiscale fluctuation-based dispersion entropy, hierarchical feature weighting, small-sample learning

## Abstract

To address information loss and imbalanced feature contributions in bearing fault diagnosis under strong noise and limited training samples, this paper proposes a diagnostic method combining modal soft-suppression reconstruction (Soft-VMD) with discriminative-ability-driven hierarchical soft weighting. Following standard variational mode decomposition (VMD), Soft-VMD retains suppressed modes with small nonzero weights, providing continuous adjustment between noise suppression and information utilization. Hierarchical soft weighting uses bootstrap-averaged Fisher estimates from training source recordings to jointly adjust the scale contributions of refined composite multiscale fluctuation-based dispersion entropy (RCMFDE) and the distance contributions of different feature domains, together with grouped parameter selection within the training set and global distance normalization. In recording-disjoint Paderborn experiments, four conditions were classified: healthy, artificially induced outer-race damage, combined inner- and outer-race damage caused by real fatigue, and artificially induced inner-race damage, using 10, 20, and 30 training windows per class, respectively. Soft-VMD improved mean accuracy over fixed hard truncation under six white-noise conditions; full hierarchical weighting improved performance over unweighted features in three small-sample scenarios. All these comparisons remained significant after Holm correction within their respective exploratory testing families. Experiments with synthetic pink noise further demonstrated the improvement of soft reconstruction over hard truncation, while both the pink-noise and CWRU leave-one-load-out experiments supported the performance gains of full weighting over unweighted features. The findings support soft reconstruction over fixed hard truncation in the Paderborn white- and pink-noise experiments, and full hierarchical weighting over unweighted features in the evaluated Paderborn and CWRU settings, providing a useful approach to diagnosis with limited training samples under these experimental conditions.

## 1. Introduction

Rolling bearings are essential components of rotating machinery systems, including electric motors, aeroengines, and gearboxes, and their operating conditions directly affect the reliability and safety of mechanical equipment. Under the combined long-term effects of alternating loads, rotational speed fluctuations, and complex transmission paths, bearings are susceptible to localized damage on the inner race, outer race, and rolling elements. When a damaged region periodically contacts other components, fault-related transient impacts typically arise in the vibration signal, exhibiting modulation, nonstationarity, and nonlinearity. Relevant reviews and benchmark studies have systematically discussed rolling-bearing impact responses, envelope analysis, and differences in diagnosis using public datasets [1]. Therefore, extracting stable condition information from vibration responses to identify bearing faults has become an important research direction in machinery condition monitoring and intelligent operation and maintenance.

However, bearing vibration signals acquired in practice are generally not ideal noise-free signals. Background equipment vibration, sensor measurement noise, structural transmission paths, installation conditions, and coupling among other mechanical components can all alter the manifestation of fault impacts in observed signals. Particularly under strong noise, weak fault components can be masked by broadband random noise, making the periodicity and modulation patterns of fault impacts difficult to observe directly. Previous studies on noise-robust feature learning and diagnosis under different load conditions have revealed the substantial influence of noise on bearing identification performance [2,3]. Noise affects not only time-domain statistics and spectral structure but also the estimation of nonlinear complexity features, thereby reducing separability among fault classes. Appropriate decomposition and reconstruction of raw vibration signals before classification are therefore important for improving the identifiability of weak fault information.

In addition to noise interference, practical fault diagnosis frequently faces limited training samples. Obtaining real fault samples generally requires equipment to be in specific abnormal conditions, while data acquisition, condition verification, and manual annotation are all costly. Reviews of machinery fault diagnosis and machine health monitoring indicate that data quality, operating-condition variations, and insufficient labeled samples remain key factors restricting the practical application of data-driven methods [4,5]. The low occurrence frequency of some fault conditions also makes it difficult to obtain sufficient and evenly distributed labeled data. With fewer training samples, classification models become more sensitive to feature redundancy, random noise, and training-set partitioning. On the one hand, noise-affected features may cause models to learn variations unrelated to fault classes [2,3]; on the other hand, excessively high feature dimensionality and numerous weakly discriminative features increase estimation difficulty under small-sample conditions. Strong noise and limited samples are therefore not independent problems: the former reduces the quality of useful fault information in individual samples, while the latter limits the classifier’s ability to identify stable patterns from data, jointly increasing the difficulty of bearing fault diagnosis.

Distinguishing anomalous information from interference has also attracted attention in broader research on state estimation. In estimating false data injection attacks in process control systems, Gajjar et al. noted that attack parameters may not be uniquely identifiable when the effects of attacks on observed data are difficult to distinguish from process disturbances or measurement noise [6]. From the perspective of anomaly identification, this study illustrates the importance of distinguishing target responses from interference and provides background for the focus of this paper on condition discrimination under noise.

For bearing fault diagnosis with limited labeled data, Wang et al. proposed a related-task-aware curriculum meta-learning (RT-ACM) framework, which adjusts knowledge utilization according to the relevance between auxiliary operating conditions and the target task and organizes meta-training through an easy-to-hard curriculum sampling strategy to support adaptation across operating conditions with limited target samples [7]. Such studies emphasize learning transferable representations from related tasks; this paper investigates improvements in condition identification with limited training samples through noisy-signal reconstruction and contribution adjustment of predefined features.

Signal decomposition is an important technical approach for suppressing noise interference and enhancing fault information. Dragomiretskiy and Zosso proposed variational mode decomposition (VMD), which adaptively decomposes a complex signal into several modes with finite bandwidths while estimating their center frequencies by formulating a constrained variational optimization problem [8]. Compared with directly extracting features from raw signals, VMD assigns signal components with different frequency structures to different modes, providing a basis for subsequent noise suppression and fault-component reconstruction. Previous studies have applied VMD to pump-bearing fault feature extraction, adaptive parameter optimization, power-information-guided decomposition, deep-network input construction, and envelope spectrum entropy feature extraction [9,10,11,12,13,14], demonstrating that VMD has become an important tool for analyzing vibration signals from rotating machinery. Intuitively, VMD separates a vibration signal into several components concentrated primarily around different frequencies, allowing their individual contributions to the reconstructed signal to be adjusted subsequently.

Existing VMD-based fault diagnosis pipelines typically select so-called fault-sensitive modes using indicators such as kurtosis, correlation coefficients, energy, or entropy, and then reconstruct the signal using the selected modes. These methods generally treat mode selection as a binary decision: a mode is either fully retained or entirely discarded. Although intuitive, this approach implicitly assumes that modes can be clearly divided into useful modes and noise modes. Under strong noise, this assumption does not always hold. VMD yields band-limited components distributed around different center frequencies rather than an absolute separation of fault information and noise. Consequently, higher-order modes may consist primarily of random noise while still containing weak impacts, modulation sidebands, or other condition-related information. Completely removing these modes may reduce noise energy but also discards residual fault information; conversely, retaining all modes may weaken the denoising effect of signal decomposition. Modal reconstruction therefore requires a trade-off between noise suppression and information preservation.

In addition to signal decomposition, multiscale complexity analysis can characterize the dynamics of nonstationary vibration signals at different time scales. Costa et al. established the fundamental framework of multiscale entropy analysis [15,16], while Chen et al. improved similarity measurement for finite-length sequences using continuous fuzzy membership functions [17]. Single-scale features generally reflect signal patterns at only one temporal resolution, whereas bearing fault responses may simultaneously contain localized impacts, periodic modulation, and slower energy variations, which are difficult to characterize adequately using single-scale statistics alone. Wu et al. successively proposed composite multiscale entropy and refined composite multiscale entropy, improving the stability of multiscale estimation by constructing multiple coarse-grained sequences with different starting points at the same scale [18,19]; Azami et al. further investigated refined composite multiscale fuzzy entropy and dispersion entropy [20,21]. In rolling-bearing diagnosis, composite multiscale fuzzy entropy (CMFE), sigmoid-based refined composite multiscale fuzzy entropy (RCMFE), and time-shift multiscale fuzzy entropy have each been combined with support vector machine (SVM) methods to extract complexity features from short sequences [22,23,24].

Coarse-graining with different starting points can be understood as changing the starting point for grouping sample points at the same scale. For example, at scale 3, every three consecutive sample points are averaged; starting from the first, second, and third sample points, respectively, produces three coarse-grained sequences at the same scale with different starting points. The refined composite construction further combines the pattern probabilities of these sequences to reduce the influence of selecting a single starting point on entropy estimation.

However, adding multiscale features does not necessarily yield a corresponding improvement in classification performance. Different scales reflect different dynamic structures and may differ in their ability to distinguish fault classes. Directly concatenating all scale features with equal weights for classification assumes that all scales have equal classification value. With limited training samples, features that are strongly affected by noise, weakly discriminative between classes, or highly redundant with adjacent scales may increase the classifier’s learning burden. Another straightforward approach is to retain only a few strongly discriminative scales, but hard scale selection also suffers from threshold sensitivity and complete information loss. Thus, similar to modal reconstruction, multiscale feature fusion requires a mechanism that continuously represents the relative importance of different scales.

This paper focuses on supervised classification of known bearing conditions: a classification model is established using labeled training windows and then used to identify the conditions of test windows whose source recordings are disjoint from the training source recordings. Envelope analysis extracts fault information from modulation structures, while deep learning, transfer learning, and few-shot learning investigate feature learning, the utilization of existing knowledge, and modeling with limited annotations [1,4,5]. For example, Shi et al. combined digital-twin-generated bearing data with a multi-scale convolutional neural network, an attention mechanism, and bidirectional gated recurrent units to support fault classification when fault samples are scarce [25]. That approach illustrates how simulated data and learned representations can support bearing diagnosis, while the present study focuses on preserving information during signal reconstruction and explicitly adjusting the contributions of predefined features under strong noise and limited training samples. He et al. investigated bearing fault diagnosis using a Siamese Vision Transformer [26]. To address the research objectives of this paper, time- and frequency-domain statistics and multiscale entropy with explicitly defined dimensionalities are used to construct features, and RBF-SVM is used for classification. This setting facilitates an explicit description of the contributions of scales and feature domains and, with a fixed classifier within each branch, a separate evaluation of the effects of modal soft reconstruction and hierarchical weighting.

Based on the above analysis, this paper introduces the concept of soft information preservation at two levels: signal reconstruction and feature fusion. Soft information preservation adjusts the contributions of different information components through continuous weights, providing smooth adjustment between full retention and complete removal. At the signal reconstruction level, the variational model and solution procedure of standard VMD remain unchanged; after obtaining the modes, small but nonzero reconstruction weights are assigned to the remaining modes. At the feature fusion level, 20 scales of refined composite multiscale fluctuation-based dispersion entropy (RCMFDE) features are extracted, and bootstrap-averaged Fisher estimates from training source recordings are used to hierarchically adjust scale contributions and the contributions of the time, frequency, and entropy domains. Scale weights, domain weights, and their parameter selection are all determined from the training data.

Although these two levels operate on signals and features, respectively, they share the same design motivation: when useful information and interference cannot be fully separated, continuously reducing the contributions of uncertain components suppresses their adverse effects while avoiding the complete loss of potentially discriminative information. Modal soft suppression primarily addresses signal quality under strong noise; scale-level and domain-level soft weighting address differences among scales within the same entropy feature and differences in contributions among heterogeneous feature domains, respectively.

To evaluate the method, recording-disjoint small-sample experiments are constructed using the Paderborn dataset. Layered validation is conducted through reconstruction ablation, four-method weighting ablation, entropy representation comparison, local parameter sensitivity, class-level analysis, and computational cost analysis, supplemented by experiments with synthetic pink noise and CWRU leave-one-load-out evaluation.

The main contributions of this paper are as follows:

1. A modal soft-suppression reconstruction strategy based on standard VMD decomposition results is proposed. During modal reconstruction, the contributions of higher-order modes are continuously reduced rather than completely removed, providing a smoother trade-off between noise suppression and preservation of potential fault information.
2. A discriminative-ability-driven hierarchical soft weighting method is proposed. Based on bootstrap-averaged Fisher estimates from training source recordings, 20 RCMFDE scales and the time, frequency, and entropy domains are jointly weighted. Domain-weight shrinkage, grouped parameter selection within the training set, and global distance normalization are combined to adjust contributions to classification distances at different levels.

The remainder of this paper is organized as follows: Section 2 presents related work and basic feature definitions, Section 3 describes the proposed method, Section 4 details the experimental settings, Section 5 analyzes the experimental results, Section 6 discusses the applicability of the method, and Section 7 concludes the paper.

## 2. Related Work

Research on bearing diagnosis with limited labeled data addresses both knowledge utilization across operating conditions and effective representation of discriminative information from limited samples. RT-ACM constructs different operating conditions as auxiliary tasks and combines task relevance with curriculum sampling to learn representations adaptable to the target task [7]. This task-level knowledge utilization and the feature-contribution adjustment in this paper operate at different levels: the former uses auxiliary tasks for meta-training and target-task adaptation, while this paper estimates the Fisher discriminative ability within the corresponding training data and continuously adjusts the distance contributions of entropy scales and different feature domains. Accordingly, the signal-processing and hierarchical weighting framework in this paper provides an interpretable feature-modeling approach for diagnosis with limited annotations.

### 2.1. VMD and Modal Reconstruction

VMD is a nonrecursive signal decomposition method based on variational optimization, capable of decomposing complex nonstationary signals into several modes with finite bandwidths and different center frequencies [8]. To address the number of modes, penalty factor, and sensitive-mode selection for bearing signals, previous studies have used envelope kurtosis, frequency-band entropy, swarm intelligence optimization, and signal power information to adaptively determine VMD parameters or modes [9,10,11,12]. Given an input signal x(t), VMD jointly estimates *K* modes ${\left\{u_{k}\left(t\right)\right\}}_{k=1}^{K}$ and their center angular frequencies ${\left\{\omega_{k}\right\}}_{k=1}^{K}$. These decomposed components are referred to as intrinsic mode functions (IMFs), with IMF*k* denoting the component $u_{k}\left(t\right)$. The corresponding constrained variational problem is

(1) $$\begin{array}{cc} \min_{\left\{u_{k}\right\},\left\{\omega_{k}\right\}}\; & \sum_{k=1}^{K}{∥\partial_{t}\left[\left(\delta \left(t\right)+\frac{j}{\pi t}\right)*u_{k}\left(t\right)\right]e^{-j\omega_{k}t}∥}_{2}^{2},\\ s.t.\; & \sum_{k=1}^{K}u_{k}\left(t\right)=x\left(t\right). \end{array}$$

where δ(t) is the Dirac delta function, *j* is the imaginary unit, ∗ denotes convolution, $\partial_{t}$ denotes differentiation with respect to time, and ${∥\cdot ∥}_{2}$ is the $L_{2}$ norm. The Hilbert kernel δ(t)+j/(πt) constructs the analytic signal, and $e^{-j\omega_{k}t}$ shifts the *k*th mode to baseband, after which the squared norm of the analytic signal gradient measures its bandwidth. Here, *t* denotes time, k=1,…,K is the mode index, *K* is the total number of modes, $u_{k}\left(t\right)$ is the *k*th mode, and $\omega_{k}$ is its center angular frequency; s.t. indicates the reconstruction constraint.

After decomposition, a general form of weighted reconstruction can be expressed as

(2) $$\hat{x}\left(t\right)=\sum_{k=1}^{K}a_{k}u_{k}\left(t\right),\;0\leq a_{k}\leq 1,$$

where $a_{k}$ is the reconstruction weight of the *k*th mode. Conventional hard selection sets $a_{k}\in \left\{0,1\right\}$, whereas continuous weighting allows a mode to participate in reconstruction at partial amplitude. In this paper, the reconstruction strategy that applies continuous modal weights after standard VMD is termed Soft-VMD, and its diagnostic performance is compared with that of hard removal of higher-order modes. In this equation, $\hat{x}\left(t\right)$ denotes the weighted reconstructed signal. Previous studies have also applied continuous weights to reconstruction after decomposition. For example, Huang et al. determined modal weights from fault-related energy ratios and performed weighted reconstruction of modal envelopes for fault diagnosis of low-speed rolling bearings [27]. This paper applies two fixed groups of amplitude coefficients to the vibration modes obtained using standard VMD: the prefix modes are fully retained, while a uniform small nonzero weight is applied to the remaining modes. This design focuses the investigation on the classification benefit of soft suppression over complete removal under the same decomposition and retention boundary; adaptive contribution adjustment driven by discriminative ability is performed through the subsequent hierarchical feature weighting.

### 2.2. Time-Domain Statistical Features

Let the reconstructed discrete vibration sequence be ${\left\{x_{i}\right\}}_{i=1}^{N}$. The definitions of the nine time-domain statistics are summarized in Table 1, where *N* is the number of sample points, $x_{p}=\max_{i}\left|x_{i}\right|$ is the absolute peak value, ${\bar{x}}_{\mathrm{abs}}=N^{-1}\sum_{i}\left|x_{i}\right|$ is the mean absolute value, and $x_{r}={\left(N^{-1}\sum_{i}\sqrt{|x_{i}|}\right)}^{2}$ is the amplitude reference for the clearance factor. Here, *i* is the sample-point index within a window, $x_{i}$ is the amplitude at the *i*th point, and μ and σ are the mean and population standard deviation of the window amplitudes, respectively. The vector $F_{T}$ denotes the nine-dimensional time-domain features, $R^{d}$ denotes the *d*-dimensional real space, and square brackets indicate concatenation in the specified order.

The resulting nine-dimensional time-domain feature vector is

$$F_{T}=\left[\mu ,\sigma ,\mathrm{RMS},\mathrm{Skew},\mathrm{Kurt},\mathrm{CF},\mathrm{SF},\mathrm{IF},\mathrm{CLF}\right]\in R^{9}.$$

### 2.3. Frequency-Domain Statistical Features

A real fast Fourier transform is applied to the real-valued sequence of length *N*, and the DC component is removed. Let $A_{k}=\left|\mathrm{RFFT}{\left(x\right)}_{k}\right|$ and $f_{k}=kf_{s}/N$, where k=1,…,M, $f_{s}$ is the sampling frequency, and *M* is the number of positive-frequency points. The six frequency-domain statistics are summarized in Table 2. Here, $x=[x_{1},\ldots ,x_{N}]$ is the input window vector, RFFT denotes the real fast Fourier transform, $A_{k}$ is the amplitude at the *k*th retained frequency point, and $f_{k}$ is its frequency; here, *k* is the frequency-point index. $F_{F}$ and $F_{TF}$ denote the six-dimensional frequency-domain features and the fifteen-dimensional concatenated time- and frequency-domain features, respectively.

The six-dimensional frequency-domain vector is denoted as

$$F_{F}=\left[\bar{A},\sigma_{A},f_{c},E_{f},f_{\mathrm{MS}},\sigma_{f}\right]\in R^{6}.$$

Concatenating it with the time-domain vector yields $F_{TF}=\left[F_{T},F_{F}\right]\in R^{15}$.

### 2.4. RCMFDE and Entropy Representation Baselines

Multiscale entropy complements conventional statistical features with complexity at different time scales [15,16]. Composite and refined composite constructions use different starting points at the same scale to reduce the influence of any single starting point [18,19,21]. This paper uses multiscale fuzzy entropy (MFE), CMFE, and RCMFE as entropy representation baselines, with fuzzy similarity measures consistent with previous fuzzy entropy studies [17,20].

For a sequence used in fuzzy entropy calculation, templates of length *m* are constructed, and the mean of each template is subtracted from that template. Let two mean-centered templates be ${\tilde{u}}_{i}$ and ${\tilde{u}}_{j}$. Their difference is measured using the Chebyshev distance:

(3) $$d_{\infty }\left({\tilde{u}}_{i},{\tilde{u}}_{j}\right)=\max_{k=1,\ldots ,m}\left|{\tilde{u}}_{i,k}-{\tilde{u}}_{j,k}\right|.$$

where i,j are template indices, *k* is the component index within a template, and ${\tilde{u}}_{i,k}$ and ${\tilde{u}}_{j,k}$ are the *k*th components of the two mean-centered templates, respectively. This distance takes the maximum absolute difference between corresponding components. The baseline implementation further uses $\exp[-{\left(d_{\infty }/r\right)}^{n}]$ to convert template distances into fuzzy similarities, where *r* is the similarity tolerance and *n* is the fuzzy exponent; self-matches are excluded from the calculation.

The RCMFDE used in this paper refers to refined composite multiscale fluctuation-based dispersion entropy, which is based on fluctuation patterns and multiscale construction [28,29]. For samples $x_{i}$, at scale τ and offset q∈{1,…,τ}, the following is constructed:

(4) $$y_{j}^{\left(\tau ,q\right)}=\frac{1}{\tau }\sum_{i=(j-1)\tau +q}^{j\tau +q-1}x_{i},\;j=1,\ldots ,\left\lfloor \frac{N-q+1}{\tau }\right\rfloor .$$

where $y_{j}^{\left(\tau ,q\right)}$ is the *j*th coarse-grained value at scale τ and starting offset *q*, τ is the number of sample points averaged in each segment, *j* is the coarse-grained sequence index, and ⌊·⌋ denotes the floor operation; here, *q* denotes only the coarse-graining starting offset.

The mapping parameters $\mu_{x},\sigma_{x}$ are computed once from the same input window and used for all its scales and offsets, so that all scales share the same within-window mapping reference. Each coarse-grained sequence is mapped to discrete classes:

(5) $$z_{j}^{\left(\tau ,q\right)}={\mathrm{clip}}_{\left[1,c\right]}\left(\mathrm{round}\left[c\Phi \left(\frac{y_{j}^{\left(\tau ,q\right)}-\mu_{x}}{\sigma_{x}}\right)+0.5\right]\right).$$

where $\mu_{x}$ and $\sigma_{x}$ are the mean and population standard deviation of the input window, respectively, Φ(·) is the standard normal cumulative distribution function, *c* is the number of discrete amplitude classes, and $z_{j}^{\left(\tau ,q\right)}\in \left\{1,\ldots ,c\right\}$ is the mapped class index; round denotes rounding to the nearest integer, and ${\mathrm{clip}}_{\left[1,c\right]}$ clips the result to [1,c].

Next, delay vectors $z_{j}^{\left(\tau ,q\right)}=\left(z_{j}^{\left(\tau ,q\right)},z_{j+d}^{\left(\tau ,q\right)},\ldots ,z_{j+(m-1)d}^{\left(\tau ,q\right)}\right)$ are constructed, where *m* is the embedding dimension and *d* is the delay, measured in sample points of the corresponding coarse-grained sequence. Differences between successive elements of each delay vector define fluctuation patterns of length m−1, with each difference taking a value in {−(c−1),…,c−1}. Denoting the relative occurrence frequency of pattern π in each offset sequence as $p_{\pi }^{\left(\tau ,q\right)}$, the probabilities are averaged before entropy is calculated:

(6) $${\bar{p}}_{\pi }\left(\tau \right)=\frac{1}{\tau }\sum_{q=1}^{\tau }p_{\pi }^{\left(\tau ,q\right)},\;\mathrm{RCMFDE}\left(\tau \right)=-\sum_{\pi :{\bar{p}}_{\pi }>0}{\bar{p}}_{\pi }\left(\tau \right)\ln{\bar{p}}_{\pi }\left(\tau \right).$$

where *m* is the embedding dimension, and *d* is the sampling interval between adjacent elements in the delay vector; each delay vector consists of *m* mapped classes sampled at intervals of *d* from the current position. π denotes a fluctuation pattern, $p_{\pi }^{\left(\tau ,q\right)}$ is its relative occurrence frequency in the coarse-grained sequence at scale τ and offset *q*, and ${\bar{p}}_{\pi }\left(\tau \right)$ is the probability averaged over all τ offsets. The sum includes patterns with positive average probability, and ln denotes the natural logarithm.

The experiments fixed $m=2,c=5,d=1,\tau_{\max}=20$, yielding nine possible fluctuation patterns per sequence and a twenty-dimensional entropy vector E. $\tau_{\max}$ is the maximum scale, and E=[RCMFDE(1),…,RCMFDE(20)] is the entropy feature vector arranged in scale order.

### 2.5. RBF-SVM

This paper uses a radial-basis-function support vector machine (RBF-SVM) for classification, in which the radial basis function (RBF) constructs a nonlinear kernel. A support vector machine establishes a decision boundary by maximizing the classification margin, with its statistical learning foundations established by Cortes and Vapnik [30]. SVM and its kernel methods have been widely applied to machinery condition monitoring and bearing fault classification [31,32]. The implementation in this paper follows the kernel SVM training and multiclass strategies summarized in LIBSVM [33]. For training samples $\left(x_{i},y_{i}\right)$, the RBF kernel maps Euclidean distances in the input space to similarities:

(7) $$K\left(x_{i},x_{j}\right)=\exp\left[-\gamma ∥x_{i}-x_{j}{∥}_{2}^{2}\right],$$

where γ>0 is the kernel-width parameter, and ${∥\cdot ∥}_{2}$ denotes the Euclidean norm. The binary decision function can be written as

(8) $$f\left(x\right)=\mathrm{sign}\left(\sum_{i=1}^{N_{\mathrm{sv}}}\alpha_{i}y_{i}K\left(x_{i},x\right)+b\right),$$

where $N_{\mathrm{sv}}$ is the number of support vectors, $\alpha_{i}$ is the dual coefficient, *b* is the bias, and $y_{i}$ is the class label. The experiments used a multiclass RBF-SVM. The Paderborn reconstruction ablation fixes C=1,γ=1/15, while the hierarchical weighting ablation fixes C=10, γ=1/35; all methods within the same branch use identical classifier settings. Hierarchical weighting uses a globally normalized square-root mapping to control the overall distance scale and adjust the relative contributions of individual feature dimensions.

In this subsection, $x_{i}$ and $x_{j}$ are the input feature vectors of two samples, and K(·,·) is the kernel similarity function; x is the sample to be classified, f(x) is the binary decision output, sign is the sign function, and the binary labels satisfy $y_{i}\in \left\{-1,+1\right\}$. C>0 is the SVM misclassification penalty parameter, controlling the trade-off between the classification margin and training error. $\alpha_{i}$ is the SVM dual coefficient and is defined separately from the VMD bandwidth penalty parameter α; f(x) in this subsection is also used separately from the scale floor *f* introduced later.

## 3. Proposed Fault Diagnosis Method

### 3.1. Overall Pipeline and Two Controlled Branches

**Figure 1:**
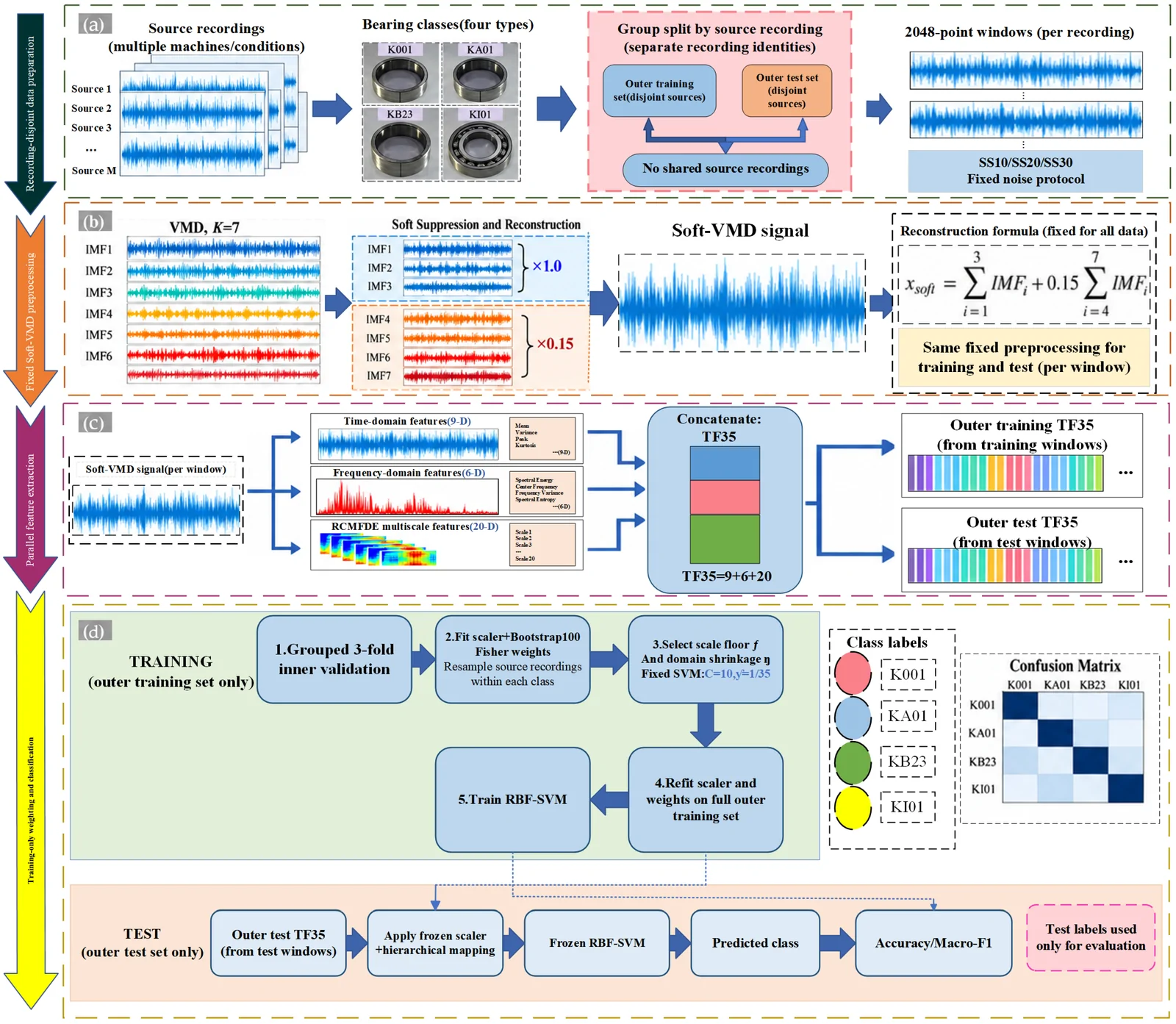
Overall pipeline of the proposed bearing fault diagnosis method. (**a**) Separation of training and test sets by source recording; (**b**) VMD modal soft-suppression reconstruction; (**c**) parallel extraction of time-domain, frequency-domain, and RCMFDE features to form a 35-dimensional fused representation; (**d**) hierarchical soft weighting and RBF-SVM classification based on grouped validation within the training set and bootstrap-averaged Fisher estimation. Standardization and weight transformations are estimated from the training data and then applied to the test set. IMF denotes an intrinsic mode function; IMF1–3 are fully retained, and IMF4–7 receive the soft-suppression coefficient λ=0.15.

The proposed method consists of VMD modal soft-suppression reconstruction and discriminative-ability-driven hierarchical soft weighting. The former performs continuous reconstruction based on standard VMD decomposition results, while the latter jointly adjusts classification distances at the scale and feature-domain levels. Nine time-domain features, six frequency-domain features, and twenty RCMFDE features are extracted from each window to form a thirty-five-dimensional fused representation. Standardization, supervised weight estimation, and parameter selection are all performed within the corresponding training data. The overall pipeline of the proposed method is shown in Figure 1, comprising, in sequence, source-recording separation, modal soft-suppression reconstruction, multidomain feature extraction, hierarchical soft weighting within the training set, and classification.

To examine the effects of the two components separately, two controlled branches are designed: the reconstruction branch uses fifteen-dimensional time- and frequency-domain statistical features (TF15) to compare Raw, VMD-All, Hard-VMD, and Soft-VMD; the hierarchical branch reuses the same Soft-VMD signals and concatenates TF15 with twenty-dimensional RCMFDE to form TF35, comparing unweighted, scale-only, domain-only, and full hierarchical weighting. For Paderborn, the two branches fix C=1,γ=1/15 and C=10,γ=1/35, respectively. A fixed classifier within each branch measures the contributions of reconstruction and weighting separately; absolute accuracy differences between branches are not treated as the net gain of a module.

### 3.2. VMD Modal Soft-Suppression Reconstruction

Standard VMD decomposes the input noisy signal according to Equation (1). The implementation uses vmdpy (version 0.2) with fixed K=7, α=200, a zero dual update step size, no fixed DC mode, uniform initialization, and a convergence tolerance of $10^{-7}$. Modes follow the original return order of the solver, and both prefix and remaining modes are defined by these indices, ensuring consistent mode correspondence across reconstruction comparisons. Here, α is the VMD bandwidth penalty parameter, controlling the strength of the modal bandwidth constraint.

With the prefix length set to L=3, reconstruction is defined as

(9) $${\hat{x}}_{\lambda }\left(t\right)=\sum_{k=1}^{L}u_{k}\left(t\right)+\lambda \sum_{k=L+1}^{K}u_{k}\left(t\right),\;0\leq \lambda \leq 1.$$

where ${\hat{x}}_{\lambda }\left(t\right)$ is the soft-suppression reconstructed signal, *L* is the number of fully retained prefix modes, and λ is the shared amplitude coefficient of the remaining K−L modes.

The main experiments fixed λ=0.15, fully retaining the prefix modes while allowing the remaining modes to participate in reconstruction with small but nonzero amplitudes. Hard-VMD corresponds to λ=0 under the same decomposition and fixed boundary, VMD-All corresponds to λ=1, and Raw is the undecomposed noisy input. All methods shared the same noise realization, and the three VMD reconstruction comparisons shared a single decomposition.

If $r_{H}=\sum_{k=4}^{7}u_{k}$, then

(10) $${\hat{x}}_{\mathrm{soft}}-{\hat{x}}_{\mathrm{hard}}=0.15r_{H},\;∥{\hat{x}}_{\mathrm{soft}}-{\hat{x}}_{\mathrm{hard}}{∥}_{2}^{2}=0.0225{∥r_{H}∥}_{2}^{2}.$$

where $r_{H}$ is the aggregate signal of the fourth to seventh intrinsic mode functions (IMF4–7), with the subscript *H* used only to identify the remaining-mode group; ${\hat{x}}_{\mathrm{soft}}$ and ${\hat{x}}_{\mathrm{hard}}$ are the reconstructions with λ=0.15 and λ=0, respectively, and the coefficient $0.0225=0.15^{2}$.

This relation specifies the amplitude-control mechanism of soft suppression: while fully retaining the first three modes, the remaining aggregate term is introduced into reconstruction at 15% of its amplitude. Hard removal is thus extended to continuous contribution adjustment, retaining part of the remaining information while limiting its influence. Its classification effect is evaluated through ablation under the same decomposition and boundary, while local variations in the fixed parameters are analyzed separately within a training subset.

### 3.3. Discriminative-Ability-Driven Hierarchical Soft Weighting

#### 3.3.1. Training-Set Standardization and Fisher Scores

Let q∈{T,F,E} denote the time, frequency, and entropy domains, with dimensionalities of 9,6,20, respectively. Each dimension is standardized using the mean and standard deviation of the corresponding training set, and test or validation data receive only this training-set transformation. Constant dimensions are transformed according to the zero-variance handling rule of StandardScaler (scikit-learn, version 1.9.0), and population variance is used. For standardized training features, the Fisher score is

(11) $$D_{q,j}=\frac{\sum_{c=1}^{J}n_{c}{\left(\mu_{c,q,j}-\mu_{q,j}\right)}^{2}}{\sum_{c=1}^{J}n_{c}\sigma_{c,q,j}^{2}+\varepsilon },\;\varepsilon =10^{-12}.$$

where $D_{q,j}$ is the Fisher score of the *j*th feature dimension in feature domain *q*, and J=4 is the number of diagnostic classes; here, c=1,…,J is the diagnostic class index, defined separately from the number of amplitude classes *c* in the RCMFDE mapping according to the respective equation. $n_{c}$ is the number of windows in class *c* in the current training data, $\mu_{c,q,j}$ and $\sigma_{c,q,j}^{2}$ are the mean and population variance of the *j*th standardized feature dimension in that class, respectively, $\mu_{q,j}$ is the mean of that dimension over all current training windows, and ε is a numerical stability constant preventing a zero denominator. Here, q∈{T,F,E} denotes the time, frequency, and entropy domains, respectively; $j=1,\ldots ,d_{q}$ is the within-domain feature index, with $d_{T}=9,d_{F}=6,d_{E}=20$. The Fisher score measures discriminative ability using between-class differences and within-class dispersion in the training features.

#### 3.3.2. Class-Stratified Source-Recording Bootstrap Averaging

A class-stratified source-recording bootstrap with B=100 resamples is used to estimate the averages of normalized Fisher statistics. In each resample, recordings are sampled with replacement within each class, with the number of sampled recordings equal to the number of training recordings in that class. All existing training windows from the sampled recordings are retained with their sampling multiplicities. The standardizer is first fitted on the current training fold, and scores are then computed by resampling these standardized features. The four ablation methods share the same resamples to maintain identical inputs for weight estimation.

For the *b*th resample, the entropy-domain scores are first min–max normalized, and the domain weights are then calculated:

(12) $$v_{s}^{\left(b\right)}=\left\{\begin{array}{cc} \frac{D_{E,s}^{\left(b\right)}-\min_{l}D_{E,l}^{\left(b\right)}}{\max_{l}D_{E,l}^{\left(b\right)}-\min_{l}D_{E,l}^{\left(b\right)}}, & \mathrm{range}\geq \varepsilon ,\\ 1, & \mathrm{range}<\varepsilon , \end{array}\right.$$

where the superscript (b) denotes the *b*th source-recording resample, b=1,…,B, and B=100 is the number of resamples; s=1,…,20 is the RCMFDE scale index, $D_{E,s}^{\left(b\right)}$ is the Fisher score at the *s*th entropy-domain scale in that resample, and $v_{s}^{\left(b\right)}$ is its normalized score. $\min_{l}$ and $\max_{l}$ range over all twenty scales, and the range is the difference between these two values.

(13) $$G_{q}^{\left(b\right)}=\max(\frac{1}{d_{q}}\sum_{j}D_{q,j}^{\left(b\right)},\varepsilon ),\;a_{q}^{\left(b\right)}=\frac{3G_{q}^{\left(b\right)}}{\sum_{r\in \{T,F,E\}}G_{r}^{\left(b\right)}}.$$

Here, $G_{q}^{\left(b\right)}$ is the mean Fisher score of domain *q* in the *b*th resample after applying the lower bound ε, and the within-domain sum ranges over $j=1,\ldots ,d_{q}$; $a_{q}^{\left(b\right)}$ is the normalized domain weight, the denominator ranges over the three feature domains, and the factor 3 ensures that the three domain weights sum to 3.

The normalized scale scores and normalized domain weights are then averaged separately:

(14) $${\bar{v}}_{s}=B^{-1}\sum_{b=1}^{B}v_{s}^{\left(b\right)},\;{\bar{a}}_{q}=B^{-1}\sum_{b=1}^{B}a_{q}^{\left(b\right)}.$$

where ${\bar{v}}_{s}$ and ${\bar{a}}_{q}$ are the arithmetic means of the normalized scale scores and domain weights from the *B* resamples, respectively.

Normalization before averaging places the estimates from different resamples on a consistent scale; degenerate scale scores are uniformly set to 1 to maintain a neutral contribution. This bootstrap is used for training-weight estimation and is performed separately from the test-recording interval estimation in the result analysis.

#### 3.3.3. Scale Floor, Domain Shrinkage, and Distance Mapping

The scale weights and the domain weights after shrinkage are defined as

(15) $$w_{s}=f+\left(1-f\right){\bar{v}}_{s},\;a_{q}=1+\eta \left({\bar{a}}_{q}-1\right),\;0\leq f,\eta \leq 1.$$

where $w_{s}$ is the final weight of the *s*th entropy scale, *f* is the scale-weight floor, η is the domain-weight shrinkage coefficient, and $a_{q}$ is the domain weight after shrinkage; η controls the extent to which the bootstrap-averaged domain weight ${\bar{a}}_{q}$ deviates from the equal-weight value of 1.

When f=1, the scale level reduces to no weighting; when η=0, the domain level reduces to no weighting. The three domain weights continue to sum to 3. Interpreting the weights as coefficients of squared distances gives

(16) $$\omega =[a_{T}1_{9},a_{F}1_{6},a_{E}w_{1},\ldots ,a_{E}w_{20}].$$

where $\omega \in R^{35}$ is the squared-distance weight vector concatenated in the order of the time, frequency, and entropy domains, $1_{9}$ and $1_{6}$ are all-ones vectors of lengths 9 and 6, respectively, and $a_{T},a_{F},a_{E}$ are the weights of the three domains; $\omega_{j}$ denotes the coefficient of the *j*th dimension after concatenation.

The feature amplitudes are multiplied by the square roots of the coefficients and normalized by the total global variance of the training set:

(17) $$g=\sqrt{\frac{\sum_{j}{\mathrm{Var}}_{\mathrm{tr}}\left(Z_{j}\right)}{\sum_{j}{\mathrm{Var}}_{\mathrm{tr}}\left(\sqrt{\omega_{j}}Z_{j}\right)}},\;F_{i,j}=g\sqrt{\omega_{j}}Z_{i,j}.$$

where $Z_{i,j}$ is the standardized feature of sample *i* in the *j*th concatenated dimension, and the mean and standard deviation used for standardization are estimated solely from the current training data; $Z_{j}$ denotes the set of values in that dimension over the training samples. ${\mathrm{Var}}_{\mathrm{tr}}$ is the population variance on the current training data, and both sums range over all 35 dimensions; *g* is the global distance normalization factor, and $F_{i,j}$ is the mapped feature value. Here, *j* is the global dimension index after concatenation and is used within its own range, separately from the within-domain index in the Fisher equation.

The same *g*, ω, and standardization parameters are applied to validation or test features. This normalization controls the overall distance scale of the training set. The corresponding RBF kernel is

(18) $$K\left(F_{i},F_{l}\right)=\exp\left[-\gamma g^{2}\sum_{j=1}^{35}\omega_{j}{\left(Z_{i,j}-Z_{l,j}\right)}^{2}\right].$$

where $F_{i}=\left[F_{i,1},\ldots ,F_{i,35}\right]$ and $F_{l}$ are the mapped features of two samples, i,l are sample indices, and γ is the RBF kernel parameter defined above; $\omega_{j}$ controls the contribution of the standardized difference in the *j*th dimension to the squared distance.

The square-root mapping makes $\omega_{j}$ directly correspond to the contribution coefficient in the squared distance, while the global factor *g* controls the overall distance scale of the training set.

#### 3.3.4. Selection Within the Training Set and Ablation Definitions

The candidate order is fixed as

f∈[1,0.8,0.6,0.5,0.4,0.3,0.2,0.1,0],η∈[1,0.75,0.5,0.25,0].

Within each outer training set, three-fold validation grouped by source recording selects parameters according to the total number of correct classifications across all out-of-fold predictions. The standardizer and bootstrap weights are estimated independently in each fold; ties are resolved by selecting the first candidate in descending order of η and then descending order of *f*. This fixed order prioritizes a larger domain-weight shrinkage coefficient and then a larger scale floor, ensuring deterministic reproducibility of tie handling. After parameter selection, the transformations are re-estimated, and the classifier is trained on the complete outer training set; the outer test set does not participate in the selection.

The four methods are defined as follows: unweighted U (f=1,η=0); scale-only S (*f* selected, η=0); domain-only D (f=1, η selected); and full SD (f,η jointly selected). The respective numbers of candidates are 1, 9, 5, and 45. The classifier parameters *C* and γ are identical, and each method’s number of weight candidates is set according to its corresponding levels. After equivalent candidates are shared, each training case requires 45×3=135 distinct inner SVM fits. The bootstrap random seed is 20261014+10000s+10r+h, where s∈{10,20,30}, *r* is the run index, h=1,2,3 is the inner-fold index, and h=0 is used for the complete outer training set. Seeds and source indices for the supplementary datasets are fixed by their respective saved protocols.

The training and prediction procedures of the full hierarchical soft weighting branch are given in Algorithm 1.
**Algorithm 1** Training and prediction of the full hierarchical soft weighting branch.**Input:** Fixed training windows, test windows, and source-recording labels; fixed preprocessing and classifier parameters**Output:** Predicted labels for the test set  1:Compute fixed Soft-VMD and TF35 features for each window without using labels  2:for each saved grouped fold within the training set do  3:    Fit the standardizer and perform 100 recording resamples using only the training portion of that fold  4:    Estimate scale scores and domain weights using Equations (12)–(14)  5:    Construct normalized square-root mappings for the fixed candidate grid and fit SVMs with fixed parameters  6:    Save the number of correct classifications in the validation portion of that fold  7:end for  8:Select f,η using the cumulative number of correct classifications and the fixed tie-breaking rule  9:Re-estimate standardization and bootstrap weights on the complete outer training set10:Map features using Equation (17) and train the SVM11:Freeze all transformations and predict the outer test set; do not reselect parameters

## 4. Experimental Settings

### 4.1. Paderborn Data and Physical Classes

The main experiments used vibration data from the Paderborn University KAt-DataCenter [34]. The sampling frequency was 64 kHz, and the window length and stride were both 2048 points, yielding nonoverlapping segments. K001, KA01, KB23, and KI01 were selected, with 200 windows retained per class, producing an 800×2048 data matrix. The classification task comprised the conditions corresponding to these four bearing codes, with source recordings used as the data-grouping unit. The class codes, damage types, and numbers of windows for the selected bearings are listed in Table 3.

### 4.2. Small Samples and Source-Recording Separation

SS denotes a small-sample scenario: SS10, SS20, and SS30 used 10, 20, and 30 training windows per class, respectively, corresponding to 40, 80, and 120 training windows in total; each test set contained 100 windows per class, totaling 400. Twenty saved splits were retained for each scenario, and all methods shared the training and test indices within the same run. For corresponding runs, the SS10 training set was contained in SS20, and SS20 was contained in SS30.

The source-recording sets for outer training and testing were disjoint, and the three-fold validation within training was also grouped by recording, preventing windows from the same original recording from entering both the current training and test sets. Standardization and supervised weights were fitted only on the current training portion, and test labels were used for final evaluation. Repeated splits reused some recordings, so the statistics were interpreted under a recording-disjoint repeated-split protocol.

### 4.3. Noise and Matched-Condition Evaluation

The main reconstruction ablation used Gaussian white noise at −5 and −10 dB, while the main hierarchical weighting ablation used −10 dB. The signal-to-noise ratio (SNR) characterizes the power ratio between the reference signal and added noise. Let the reference window be x(n) and the base noise be z(n); then,

(19) $$\begin{array}{cc} P_{x} & =N^{-1}\sum_{n}x{\left(n\right)}^{2},\;P_{z}=N^{-1}\sum_{n}z{\left(n\right)}^{2},\\ v(n) & =z\left(n\right)\sqrt{\frac{P_{x}}{10^{{\mathrm{SNR}}_{t}/10}P_{z}}},\;y\left(n\right)=x\left(n\right)+v\left(n\right). \end{array}$$

where n=0,…,N−1 is the discrete sample index within a window, *N* is the window length, and $P_{x}$ and $P_{z}$ are the finite-window average powers of the reference signal and base noise, respectively; ${\mathrm{SNR}}_{t}$ is the target SNR in dB, v(n) is the scaled added noise, and y(n) is the final noisy signal. All sums range over every sample point within the window.

The white-noise seed for each window was fixed to its saved sample index; all methods shared the noise for the same window. The reference x(n) was the measured window before synthetic noise was added, and the target SNR was determined from its finite-length power.

Additional noise validation used synthetic pink noise at −10 dB. The nonzero-frequency FFT amplitudes of Gaussian noise were shaped by $1/\sqrt{f}$, the DC term was set to zero, and the mean was removed after inverse transformation. The finite-window power was then matched using Equation (19). Three fixed noise realizations yielded 3×800=2400 feature sets. Each realization was evaluated using the original 60 outer cases; models were fitted on the corresponding pink-noise training sets, and hierarchical parameters were selected in their inner folds. Training and testing used matched noise types and intensities, and the three fixed noise realizations were evaluated separately and then averaged with equal weights. Here, f>0 denotes the frequency of an FFT frequency point and is defined separately from the scale floor *f* in hierarchical weighting according to the respective expression.

### 4.4. CWRU as a Second Dataset and Load Separation

The second dataset used CWRU drive-end vibration data [35]. Four classes were selected: healthy, inner-race fault, rolling-element fault, and outer-race fault at the six o’clock position, with a fault size of 0.007 inches. Each class included four load conditions, 0, 1, 2, and 3, with one source recording per condition, totaling sixteen recordings. The healthy recordings had an original sampling rate of 48 kHz and were first downsampled to 12 kHz by polyphase resampling with antialiasing filtering applied to the entire recording; the fault recordings remained at 12 kHz. The first 29 nonoverlapping windows of 2048 points were taken from each recording, yielding 116 windows per class and 464 windows in total.

A four-fold leave-one-load-out evaluation was used: all four test recordings in a fold came from the held-out load, totaling 116 test windows; training windows were sampled only from the other three loads, with SS10/20/30 using 10/20/30 training windows per class, respectively, and each scenario repeated 20 times. The three inner training folds were separated by the remaining three loads, yielding 4×3×20=240 outer cases. This evaluated the applicability of the method under disjoint training and test loads.

CWRU also used fixed K=7,α=200,λ=0.15,L=3. The reconstruction branch retained the two evaluated settings of C=1 and C=10, with γ=1/150, and reports both white-noise intensities in full; the hierarchical branch retained C=10,γ=1/35. Models were trained separately on the two datasets; both *C* settings in the reconstruction branch are fully reported to show the effects of changes in the classification protocol. The feature configurations, classifier parameters, and white-noise intensities for the two main branches and the supplementary CWRU experiments are summarized in Table 4.

### 4.5. Evaluation Metrics and Statistical Methods

Accuracy and macro-averaged F1 were the primary metrics, and the standard deviation across twenty splits used the population definition (ddof=0). F1 was first macro-averaged over the four classes within each run and then summarized across runs. Percentage points denote the difference between two accuracy percentages, rather than a relative percentage.

Exploratory paired statistics for accuracy followed the saved two-sided Wilcoxon signed-rank tests, with pairs formed from differences in the integer numbers of correct classifications within the same run, zero differences removed, and a normal approximation used. The first main family comprised six Soft–Hard comparisons across two SNRs and three scenarios; the second main family comprised nine comparisons of SD against U, S, and D across three scenarios. Holm correction was applied separately to each family at a threshold of 0.05. In the effect size $d_{z}=\bar{\Delta }/s_{\Delta }$, the standard deviation of the differences used the sample definition (ddof=1), and better/equal/worse counts are also reported.

Here, Δ denotes the paired difference between the accuracy of the evaluated method and that of the corresponding comparator in the same run, $\bar{\Delta }$ is the mean of the twenty paired differences, $s_{\Delta }$ is their sample standard deviation, and $d_{z}$ is the paired standardized effect size. ddof is the number of degrees of freedom subtracted when calculating variance; B/E/W denote the numbers of runs in which the evaluated method is better than, equal to, or worse than the comparator among the twenty runs, respectively, and Holm *p* denotes the corrected *p* value within the corresponding family.

Entire test source recordings were further resampled by class 5000 times to calculate conditional marginal 95% percentile intervals for paired accuracy differences. The sampling multiplicity of the same recording was shared across runs, methods, and conditions, while models, splits, and parameters remained frozen.

The hierarchical branch fixed C=10 to control the classifier settings and examine feature-contribution adjustment. The four methods used weight grids corresponding to their respective structures, resulting in different total search budgets.

### 4.6. Reproducibility Settings

The fixed VMD settings were $K=7,\alpha =200,\tau_{\mathrm{dual}}=0,\mathrm{DC}=0,\mathrm{init}=1,\mathrm{tol}=10^{-7}$; the first three returned modes were fully retained, and the remaining modes received a weight of 0.15. RCMFDE used m=2,c=5,d=1, a maximum scale of 20, and natural logarithms. The MFE/CMFE/RCMFE baselines retained the definitions $m=2,n=2,r=0.15\sigma_{y}$ from the original feature implementation. The hierarchical branch used Bootstrap100 and the fixed grid and tie-breaking order for Equation (15).

Here, $\tau_{\mathrm{dual}}$ is the VMD dual update step size, DC is the switch specifying whether a DC mode is fixed, init is the initialization flag, and tol is the convergence tolerance; DC=0 indicates that no DC mode is fixed, and init=1 indicates uniform initialization of center frequencies. In the fuzzy entropy baselines, *m* is the template embedding dimension, *n* is the exponent of the fuzzy similarity function, *r* is the similarity tolerance, and $\sigma_{y}$ is the population standard deviation of the input reconstructed window; the tolerance was determined from that window and remained constant across all its scales and offsets. Here, n,r are fuzzy entropy parameters, used separately from the sample index in the noise equation and the run index in the training random seed according to context.

The recorded environment for computational cost testing consisted of an Intel Core Ultra 5 125H CPU (Intel Corporation, Santa Clara, CA, USA), Windows 11, Python 3.12.3, NumPy 2.5.2, SciPy 1.18.1, scikit-learn 1.9.0, and vmdpy 0.2.

## 5. Experimental Results and Analysis

### 5.1. Overall Results of the Two Controlled Ablations

The two controlled ablations were conducted within their respective branches with fixed classifiers. The reconstruction branch evaluated Raw, VMD-All, Hard-VMD, and Soft-VMD using TF15, while the weighting branch evaluated U, S, D, and SD using the same Soft-VMD TF35 features. The corresponding results and paired statistical comparisons are presented in the following subsections.

### 5.2. Improvement of Soft-VMD over Fixed Hard Truncation

Table 5 presents the complete results of the four reconstruction methods under the two white-noise conditions. Soft-VMD achieves higher mean accuracy than Raw, VMD-All, and fixed Hard-VMD in all six scenarios. Its gains over Hard-VMD range from 1.1875 to 3.3375 percentage points, indicating that, for this dataset and protocol, retaining the remaining modes with small weights improves classification performance over their complete removal.

All six paired Soft–Hard comparisons remain significant after Holm correction (Table 6), with paired effect sizes $d_{z}$ of 0.786–1.855 and conditional recording-interval lower bounds above zero. The means, effect sizes, and recording intervals support the improvement of soft suppression over fixed hard truncation under this protocol from different perspectives; differences across individual runs are fully reported by the B/E/W counts. Complete macro-averaged F1 results are provided in Appendix A Table A1.

### 5.3. Four-Method Ablation of Hierarchical Soft Weighting

**Figure 2:**
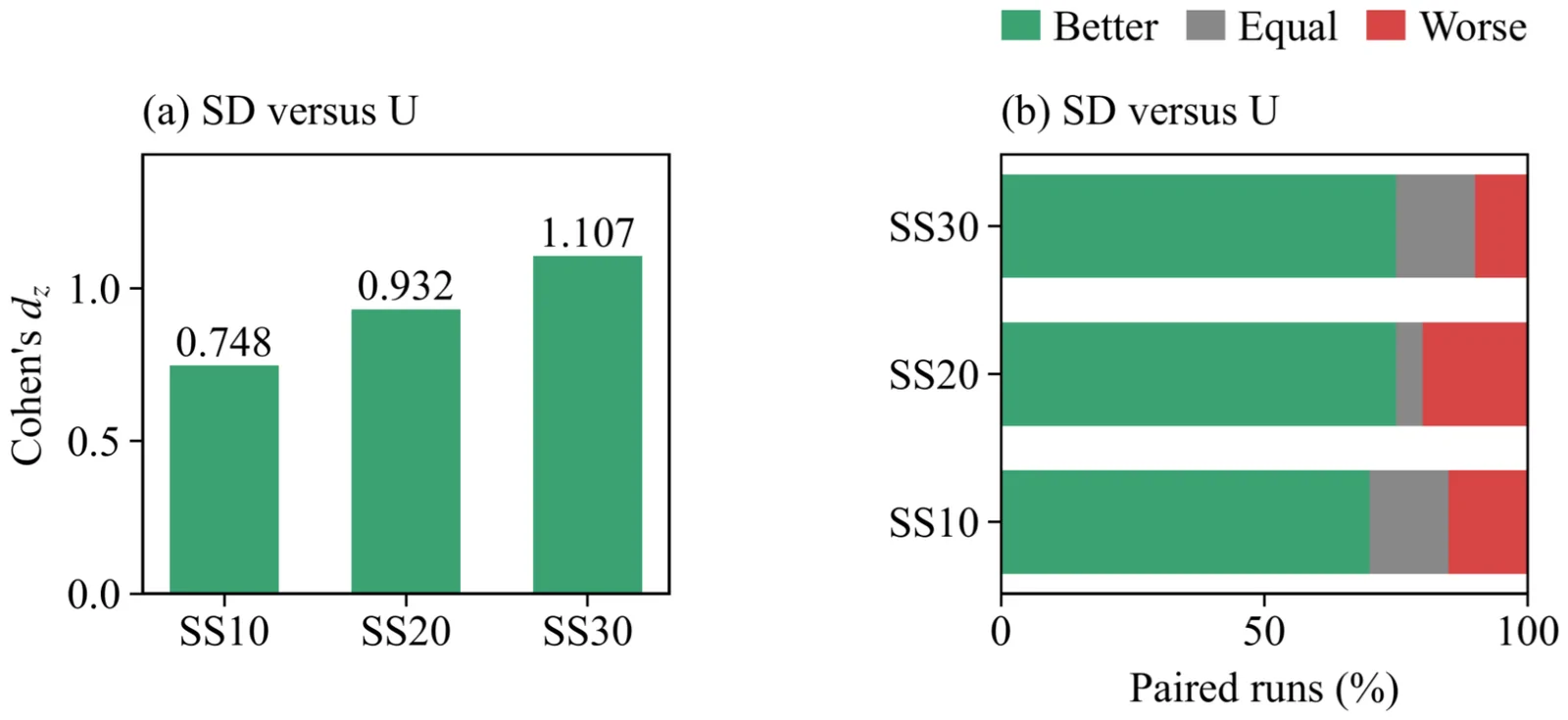
Paired statistical evidence for full hierarchical weighting (SD) relative to unweighted features (U) under Paderborn white noise at −10 dB, using the same Soft TF35 features and C=10,γ=1/35. (**a**) Paired effect size $d_{z}$; (**b**) proportions of better, equal, and worse accuracy across twenty paired runs. Complete accuracy and macro-averaged F1 results are reported in Table 7.

Table 7 compares the accuracy and macro-averaged F1 of U, S, D, and SD in the current Bootstrap100 implementation. Figure 2 complements these results with paired effect sizes and better/equal/worse proportions for SD relative to U. The full method achieves mean accuracies of 66.5250%, 71.1500%, and 73.2625% under SS10, SS20, and SS30, respectively, improving over unweighted features by 1.5500, 1.9625, and 2.2375 percentage points, respectively.

As shown in Table 8, all nine ablation comparisons yield positive mean accuracy differences, and six remain significant after Holm correction within the original family of nine comparisons. Specifically, SD versus U remains significant in all three scenarios, SD versus both single-level methods remains significant under SS20, and SD versus D also remains significant under SS30. These results support the fusion benefits of the full hierarchical mechanism. The gains for SS10 versus S, SS10 versus D, and SS30 versus S are 0.6000, 0.8000, and 0.4750 percentage points, respectively; all three corrected *p* values are 0.220824, indicating numerical improvements.

### 5.4. Comparison of Entropy Representations with the Same Classifier

**Figure 3:**
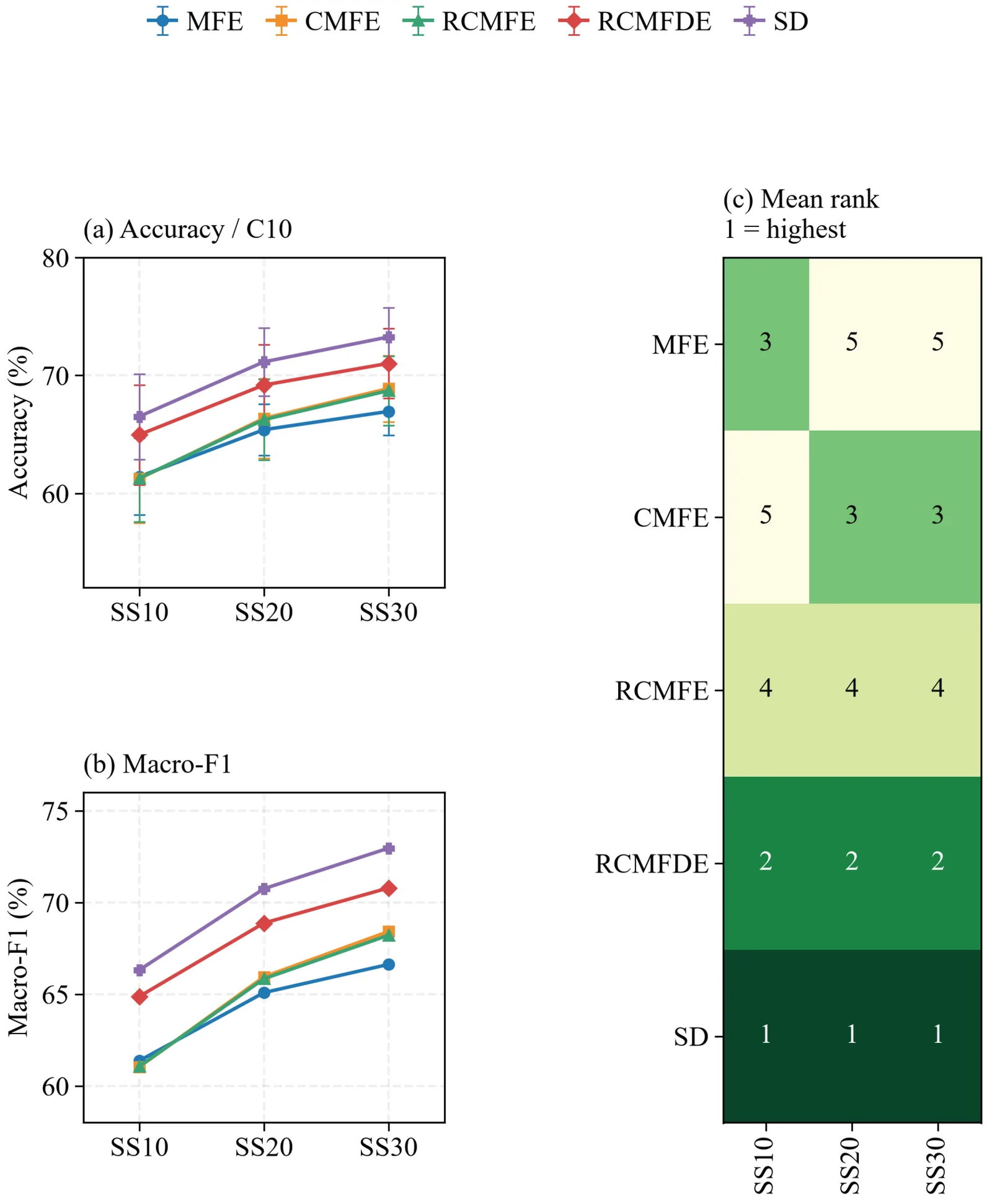
Fixed-classifier comparison of five feature schemes under white noise at −10 dB. (**a**) Mean accuracy and population standard deviation; (**b**) macro-averaged F1; (**c**) ranking by mean accuracy. In panels (**a**,**b**), blue, orange, green, red, and purple represent MFE, CMFE, RCMFE, equally weighted RCMFDE, and SD, respectively. In panel (**c**), darker green indicates a better rank, with 1 denoting the highest rank, whereas lighter yellow indicates a worse rank. All methods share Soft-VMD, TF15, and classifier settings, while the full method additionally uses hierarchical weighting within the training set.

MFE, CMFE, RCMFE, equally weighted RCMFDE, and the full method were compared using the same Soft-VMD, TF15, 20-dimensional entropy features, and fixed C=10, γ=1/35 (Table 9 and Figure 3). The full method achieved the highest mean accuracy and macro-averaged F1 in all three scenarios.

This experiment provides a representation-level comparison using the same Soft-VMD, TF15, entropy feature dimensionality, and classifier, demonstrating the mean performance of the combination of RCMFDE and hierarchical fusion.

### 5.5. Weight Selection and Local Sensitivity of Reconstruction Parameters

**Figure 4:**
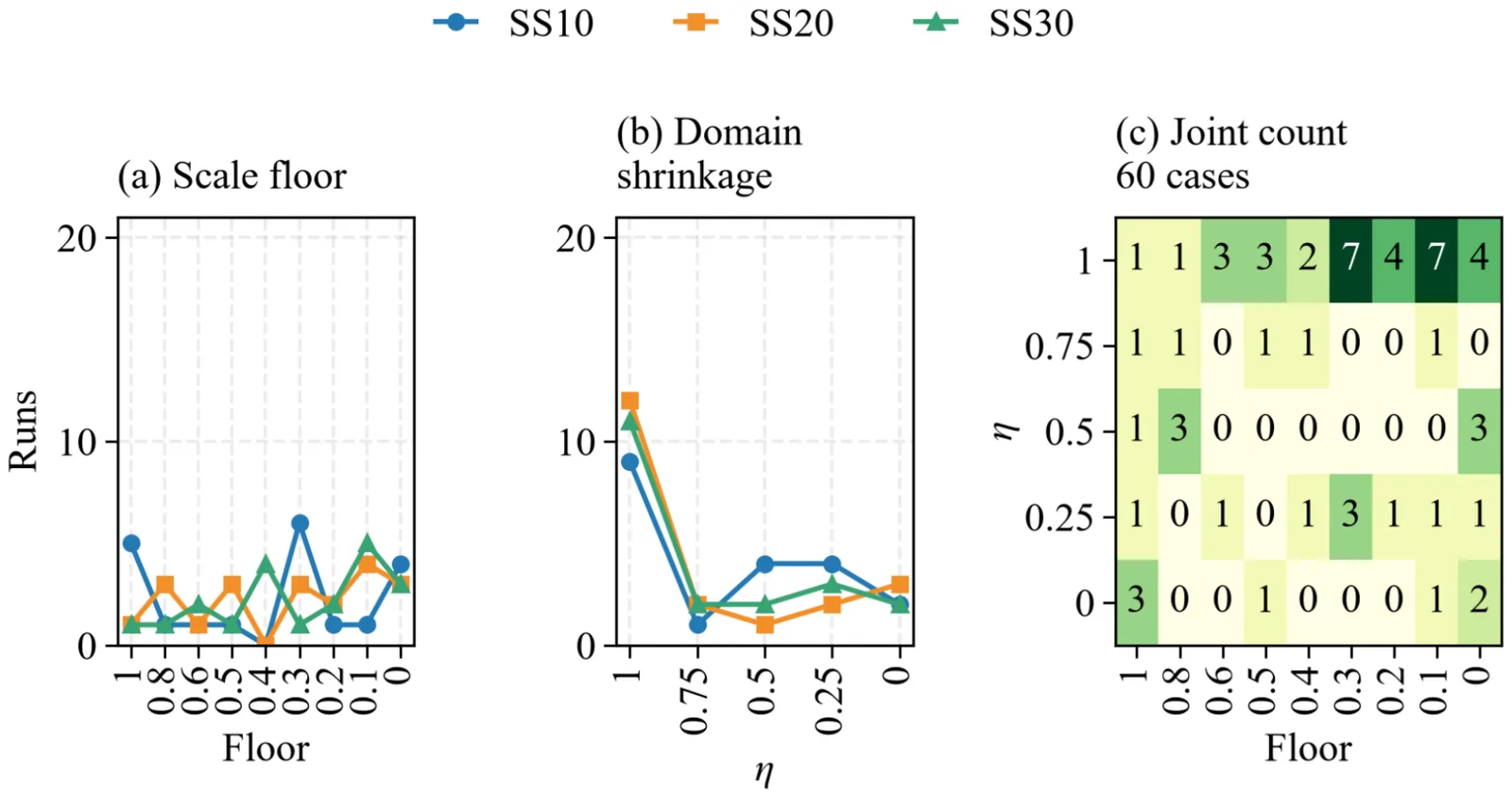
Parameter-selection frequencies within the training set for the full Bootstrap100 method. (**a**) Scale floor; (**b**) domain-weight shrinkage coefficient; (**c**) joint counts across sixty cases. In panels (**a**,**b**), blue, orange, and green represent SS10, SS20, and SS30, respectively. In panel (**c**), darker green indicates higher selection counts, whereas lighter yellow indicates lower counts; the numbers in the cells give the corresponding counts. Data are taken from the frozen parameter-selection table with fixed C=10,γ=1/35; frequencies describe selection behavior within the training set.

Figure 4 summarizes the selection frequencies of the scale floor and domain-weight shrinkage coefficient for the full method across sixty outer training cases. With a fixed grid that includes neutral configurations, the model adjusts the contributions of the two levels according to validation within the training set, allowing joint weighting as well as equal weighting at either level.

Reconstruction-parameter diagnostics are confined to the one hundred and twenty outer training windows from the first Paderborn SS30 run. Three-fold grouping by recording produces out-of-fold predictions, with only one factor among λ, the retained prefix, *K*, or α changed at a time. Complete candidate results are provided in Appendix A Table A2. At −5 dB, the central setting achieves 90.8333% accuracy, matched by λ=0.2,0.3; at −10 dB, the central setting achieves 69.1667%, compared with 70.0000% for λ=0.5, 70.8333% when retaining two modes, and 74.1667% for K=8. Conversely, K=6 yields 60.0000%, and retaining four modes yields 63.3333%. These results demonstrate parameter responses at different noise intensities, provide local supplementary evidence on the effects of the fixed settings, and suggest that the number of modes and the retention boundary deserve attention.

### 5.6. Class-Level Performance

**Figure 5:**
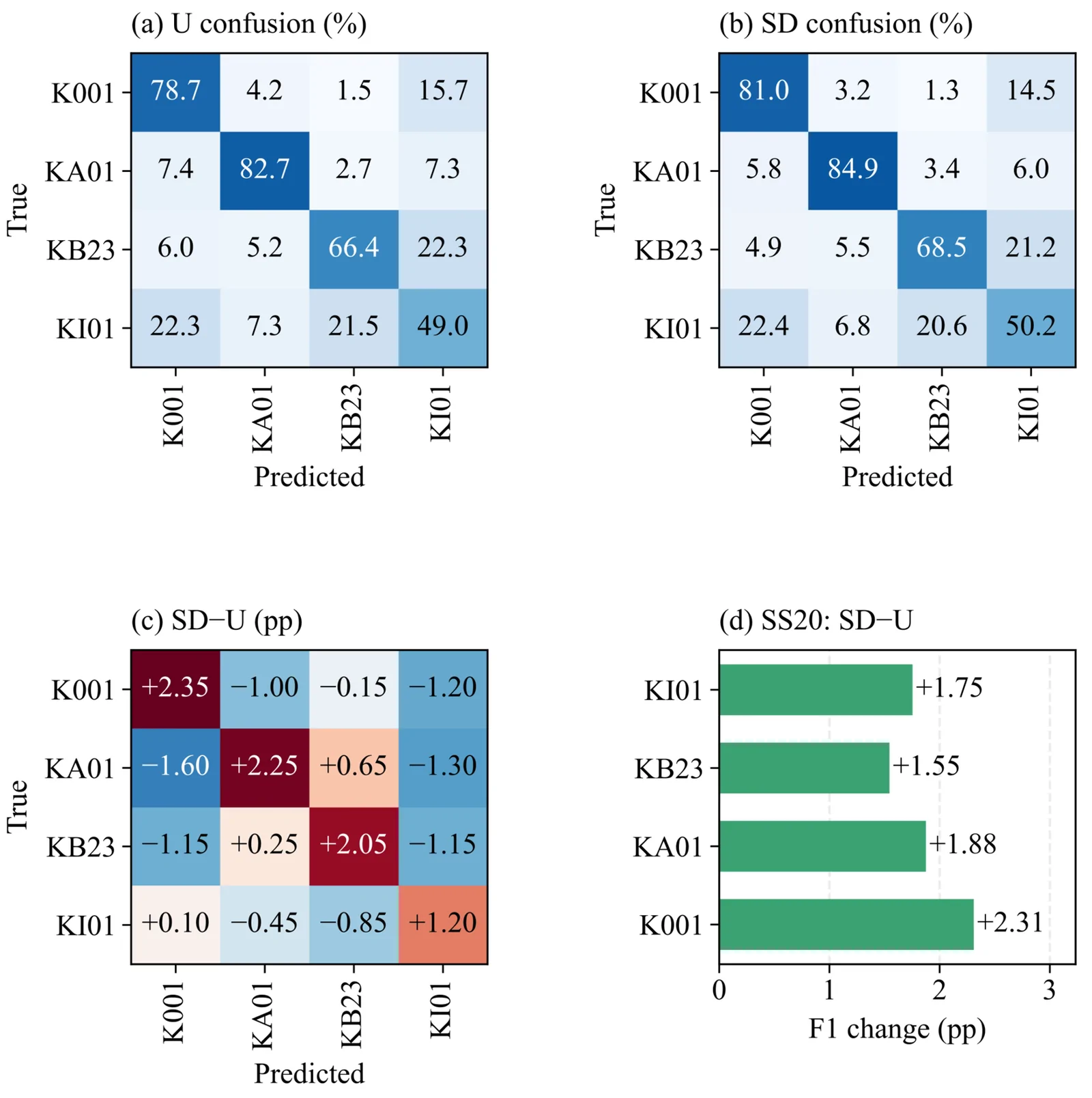
Class-level results under white noise at −10 dB, SS20, and a common C10 protocol. Mean row-normalized confusion matrices for (**a**) U and (**b**) SD; (**c**) the complete signed SD–U difference; (**d**) class-specific F1 differences calculated within each run and then averaged. In panels (**a**,**b**), darker blue indicates a higher percentage. In panel (**c**), red and blue indicate positive and negative SD–U differences, respectively, with stronger color intensity indicating larger absolute differences. In panel (**d**), green bars indicate positive class-specific F1 differences. The class order in panels (**a**–**c**) is K001, KA01, KB23, and KI01; KB23 is not the rolling-element class in CWRU.

Figure 5 uses the mean of the confusion matrices normalized by row separately for all twenty SS20 runs, without selecting the best run. Compared with U, SD improves mean recall for K001, KA01, KB23, and KI01 by 2.35, 2.25, 2.05, and 1.20 percentage points, respectively; the class-specific F1 scores improve by approximately 2.31, 1.88, 1.55, and 1.75 percentage points, respectively. Improvements are distributed across all four classes rather than depending entirely on a single class.

### 5.7. Supplementary Validation with Pink Noise

Table 10 retains all eight comparisons from the two branches, with values calculated as equal-weight averages of the twenty-run means for the three fixed noise realizations. Across the three scenarios, Soft-VMD improves over fixed Hard-VMD by 6.0000, 5.9875, and 5.5333 percentage points, respectively; full weighting improves over U by 3.5292, 4.3667, and 4.0458 percentage points, respectively.

**Figure A1:**
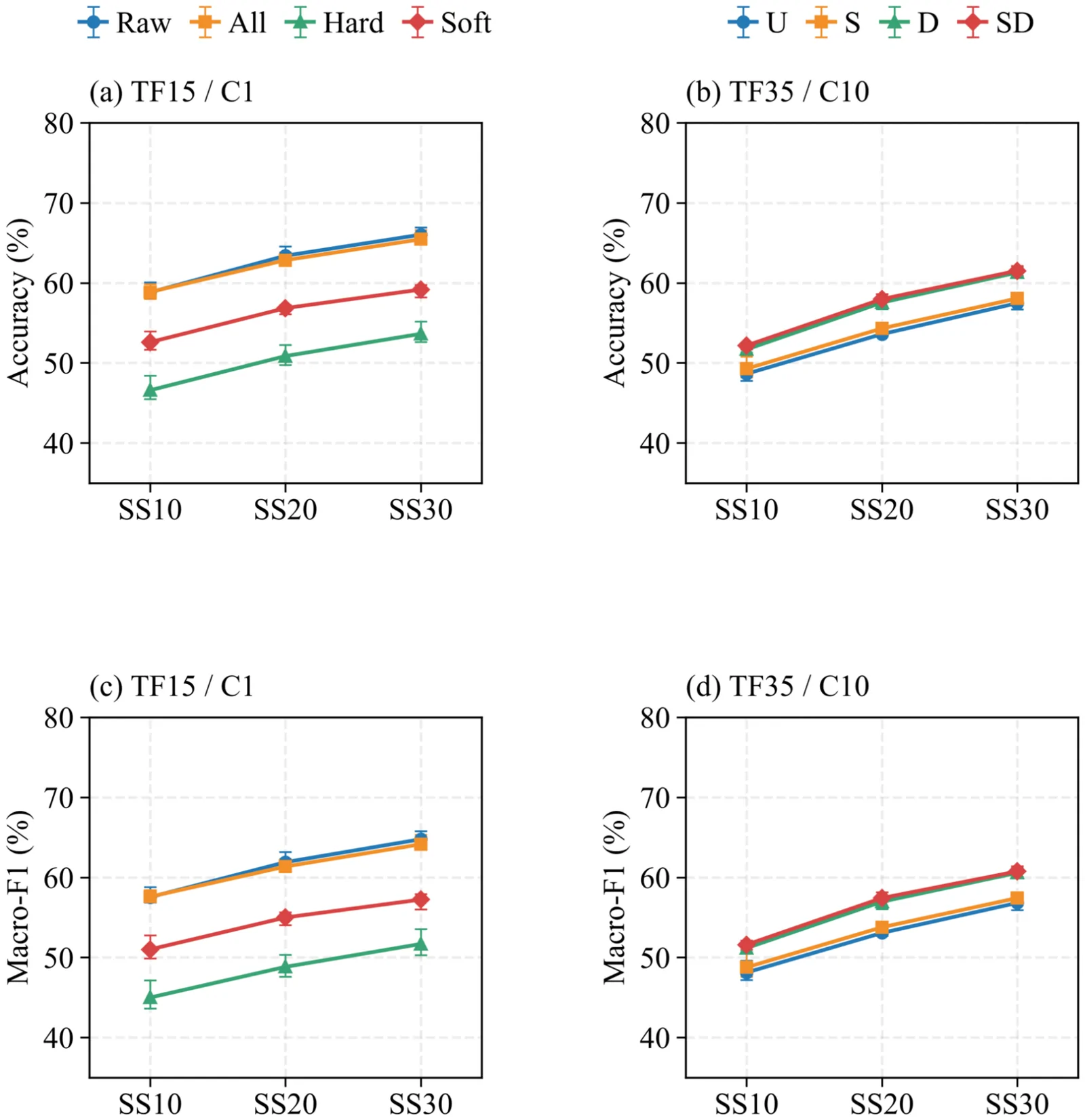
Accuracy and macro-averaged F1 of all eight methods under pink noise at −10 dB.

Under pink noise, the mean differences across realizations between full weighting and all three comparators are positive; the mean gain over D is 0.2167–0.4833 percentage points. The improvement of Soft-VMD over hard truncation has a consistent direction across the three fixed noise realizations. Complete results for all eight methods are provided in Table 10 and Appendix A Figure A1. As condition-dependent differences, Raw and VMD-All achieve higher accuracy than fixed Soft-VMD, and SD is lower than D for one noise realization under SS20; these results indicate that the benefits of different preprocessing methods and weighting levels vary with noise structure.

### 5.8. CWRU Load-Disjoint Validation

**Figure 6:**
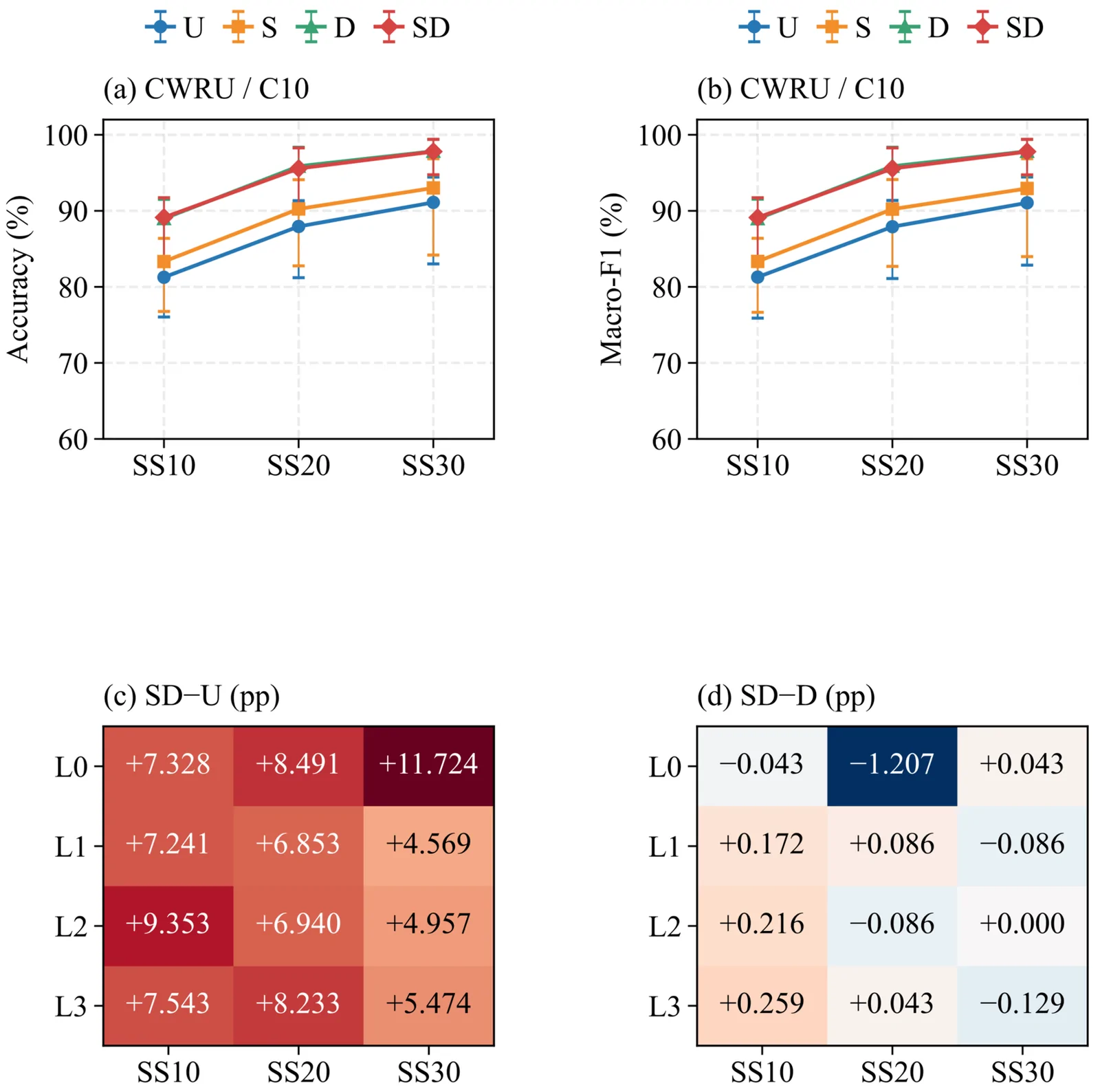
CWRU leave-one-load-out evaluation, all using TF35 and C=10,γ=1/35. (**a**) Accuracy; (**b**) macro-averaged F1; accuracy differences for (**c**) SD–U and (**d**) SD–D at each load. In panels (**a**,**b**), blue, orange, green, and red represent U, S, D, and SD, respectively. In panels (**c**,**d**), red and blue indicate positive and negative accuracy differences, respectively, with stronger color intensity indicating larger absolute differences within each panel. Points are equal-weight averages of the means for the four loads, and error bars indicate their minimum-to-maximum ranges, not confidence intervals.

Figure 6 and Table 11 present equal-weight averages of the means for the four held-out loads. Full weighting improves over U by approximately 7.8664, 7.6293, and 6.6810 percentage points under SS10, SS20, and SS30, respectively, reaching mean accuracies of 89.13%, 95.57%, and 97.79%. Results on the second dataset further support the benefits of discriminative-ability-based weighting over equal-weight concatenation. The domain level contributes strongly on this dataset: SD is slightly higher than D under SS10 but lower by approximately 0.2910 and 0.0431 percentage points under SS20 and SS30, respectively, indicating condition-dependent differences in the additional effect of the scale level.

**Figure A2:**
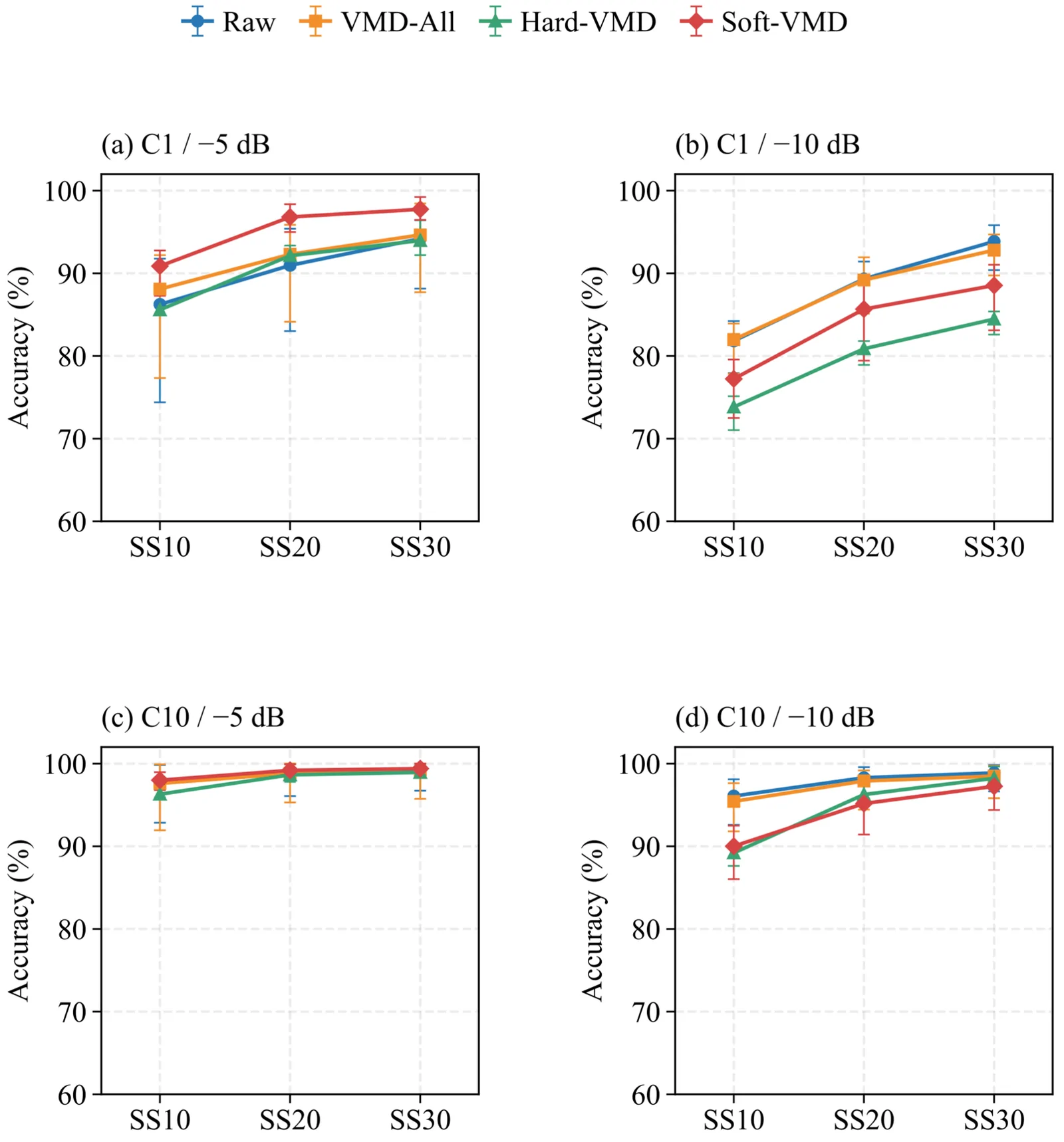
Complete accuracy of the CWRU reconstruction branch, including both *C* settings, both SNRs, and all four methods.

**Figure A3:**
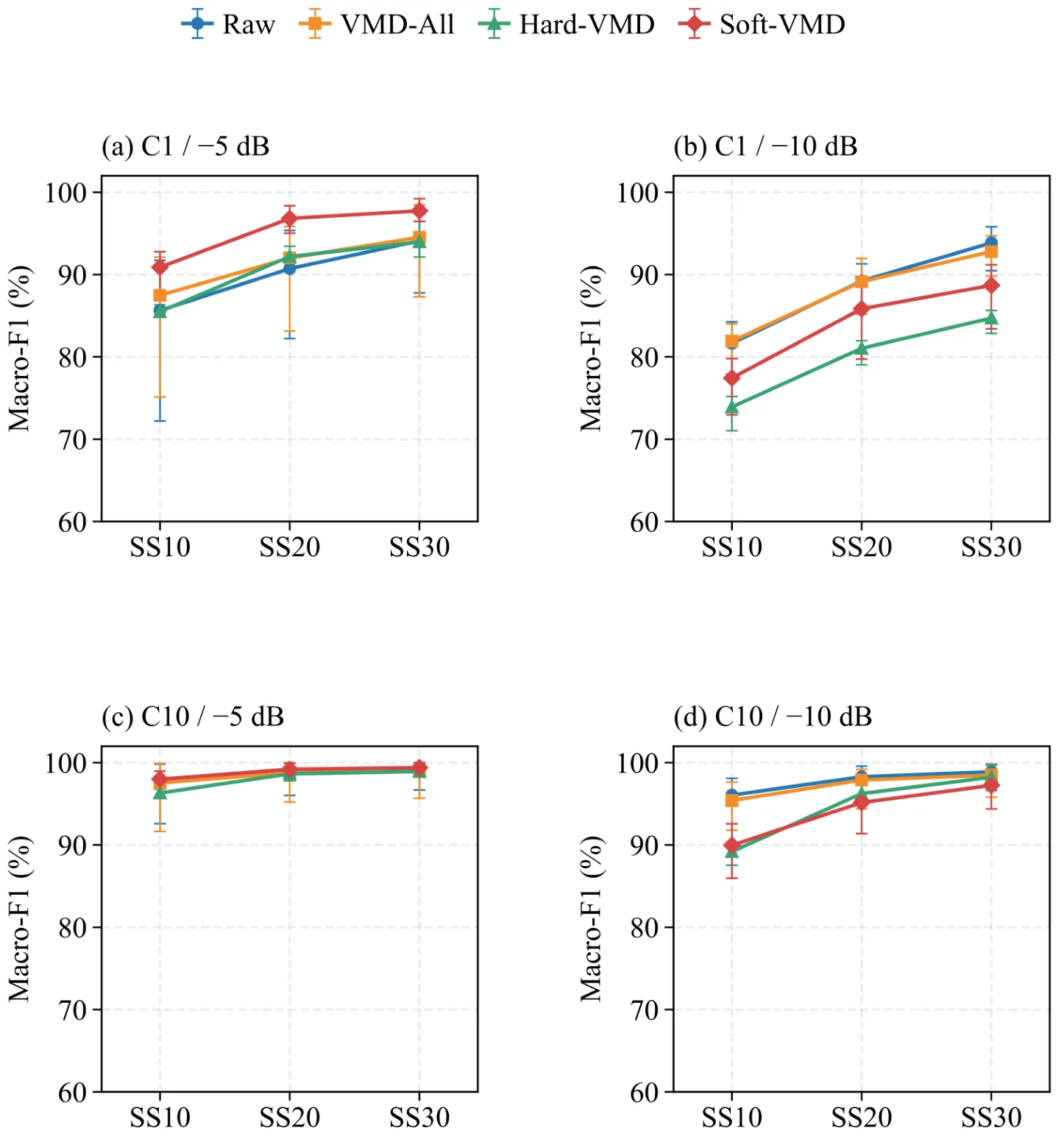
Complete macro-averaged F1 for fixed reconstruction on CWRU.

Results for the reconstruction branch with both *C* settings, both noise intensities, and all four methods are provided in Appendix A Figure A2 and Figure A3 and Table A3. At C=1,−5 dB, Soft-VMD achieves the highest mean accuracy among the four methods in all three small-sample scenarios; at C=1,−10 dB, it remains higher than fixed Hard-VMD but lower than Raw. The complete results for both classifier settings jointly demonstrate the interaction between reconstruction and the classifier.

### 5.9. Computational Cost Analysis

**Figure 7:**
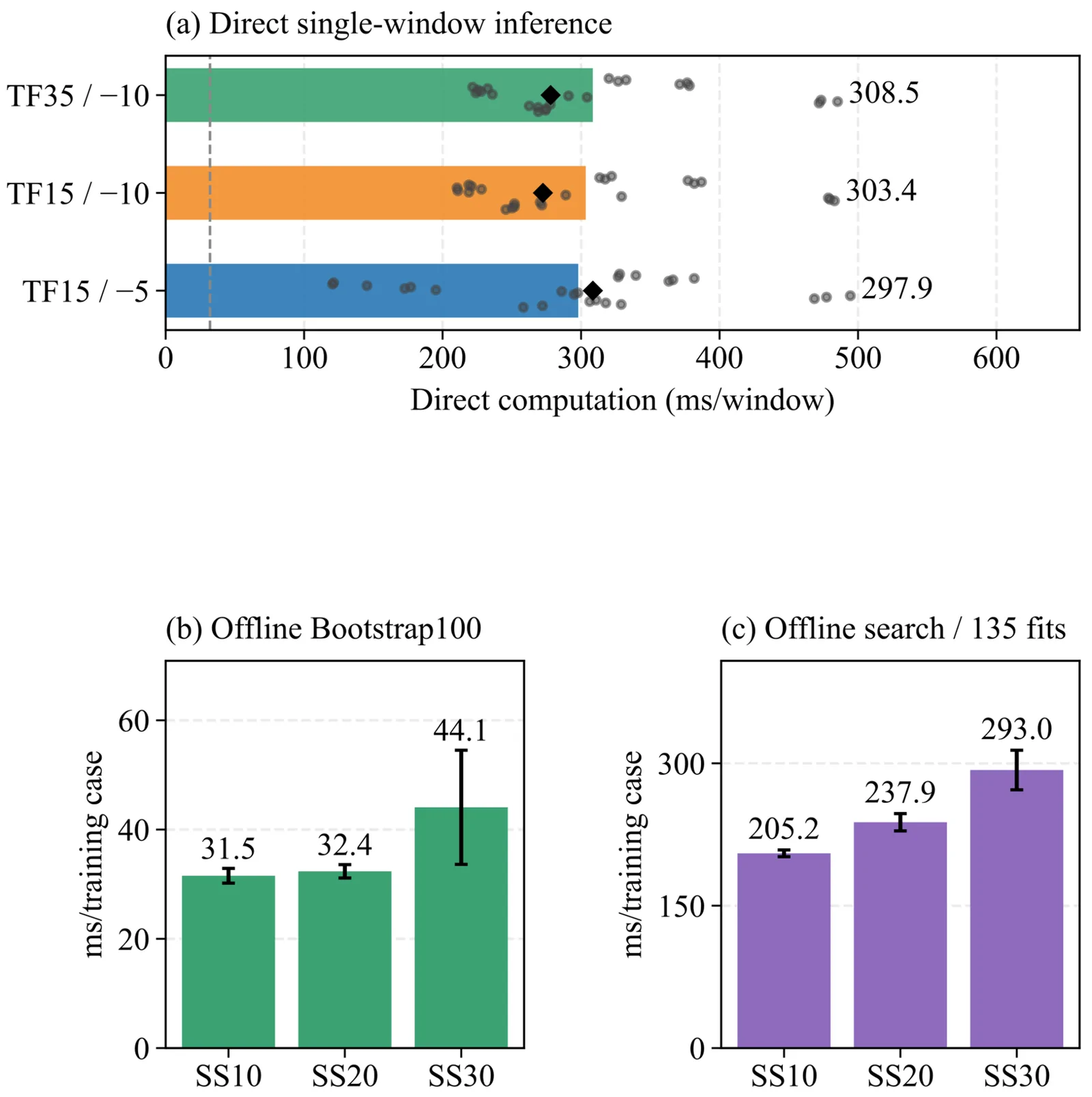
Runtime of the current CPU implementation. (**a**) Directly measured single-window end-to-end time: colored horizontal bars indicate the means, gray circular markers show all twenty-four individual measurements per configuration, and black diamond-shaped markers indicate the medians. The vertical dashed reference line at 32 ms denotes the excluded acquisition duration. (**b**) Bootstrap100 preparation and (**c**) computational replay of the search within the training set with 135 shared fits, both in ms/training case.

Figure 7 and Table 12 separate directly measured single-window end-to-end time from offline training preparation costs. Eight predefined training windows are each repeated three times using the frozen model from the first SS30 run, with an already acquired noisy window as input and a predicted label as output. The mean times for the first branch at −5 and −10 dB are 297.9394 and 303.3888 ms/window, respectively; the full branch requires 308.5027 ms/window at −10 dB. Means, medians, and all measurement points are displayed separately.

Bootstrap100 preparation averages 31.54–44.08 ms/training case across the three training sizes, including standardization and transformation of four hundred rows of evaluation features; computational replay of the shared inner search requires 205.20–292.97 ms/case, excluding feature extraction and result-table management. Weights are estimated during training, and the bootstrap is not repeated when predicting a new window. Scaling, mapping, and single-window prediction on cached features take an average of 0.1627 ms.

The current complete pipeline has a mean single-window computation time of approximately 0.31 s, providing a practical cost reference for fault analysis that permits the corresponding processing latency. Timing excludes acquisition, noise generation, file I/O, imports, and validation. A 2048-point window takes 32 ms to acquire at 64 kHz.

### 5.10. Integrated Interpretation of Statistical Evidence

As summarized in Table 6 and Table 8, all six Soft–Hard comparisons in the reconstruction branch yield positive mean differences and remain significant after Holm correction within their respective family; in the hierarchical branch, SD–U remains significant in all three scenarios, and three additional comparisons against single-level controls remain significant. The two sets of conditional recording intervals further illustrate the difference distributions of frozen predictions. Together with pink-noise experiments, the second dataset, and class-level analysis, the experiments provide complementary evidence of improvements at the signal-reconstruction and feature-fusion levels.

## 6. Discussion and Applicability of the Method

Building on existing VMD and multiscale feature frameworks, this paper introduces improvements in signal reconstruction and supervised fusion. Fixed soft reconstruction replaces complete removal with continuous contributions, while hierarchical weighting further adjusts the relative roles of scales and feature domains according to training data. Classification ablations directly examine these improvements, while training-subset parameter analysis, class-level results, and runtime provide additional information on parameter responses, the distribution of improvements, and computational costs, respectively.

The Paderborn white-noise results demonstrate improvements of Soft-VMD over fixed hard truncation and of full hierarchical weighting over unweighted features, supported by Holm-corrected comparisons within the respective testing families. The pink-noise experiments further support soft reconstruction over fixed hard truncation, while both the pink-noise and CWRU experiments support full weighting over unweighted features. Together, these results establish the benefits of the two components within the evaluated noise conditions, sample sizes, and comparison settings. The contributions of scale- and domain-level weighting vary across datasets and sample sizes, providing guidance for selecting feature-fusion settings for a given diagnosis task.

This study uses recording-disjoint and load-disjoint protocols, and the statistics are positioned as exploratory analyses under repeated splits with shared recordings. The conditional intervals do not include uncertainty from retraining, parameter selection, or method development. Parameter diagnostics are post hoc local analyses, the fixed classifiers do not represent optimal tuning for each method, and weight-search budgets vary with the structure. Future work can include comparisons with closely related soft reconstruction, supervised weighting, and data-driven mode selection within the training set using independent bearings, real industrial noise, and prespecified protocols and combine fault characteristic frequency analysis with engineering acceleration to extend the applicability of the method.

## 7. Conclusions

This paper proposed a bearing fault diagnosis method combining VMD modal soft-suppression reconstruction with discriminative-ability-driven hierarchical soft weighting. Soft-VMD continuously adjusts the contributions of remaining modes after standard decomposition. Hierarchical soft weighting jointly adjusts the distance contributions of scales and feature domains through bootstrap-averaged Fisher estimates from training source recordings, a scale floor and domain shrinkage, and a globally normalized square-root mapping.

In the recording-disjoint Paderborn white-noise experiments, Soft-VMD improved mean accuracy over fixed Hard-VMD by 1.19–3.34 percentage points under six conditions; full hierarchical weighting improved over unweighted features by 1.55–2.24 percentage points across three small-sample scenarios. All these comparisons remained significant after Holm correction within their respective exploratory testing families. All nine mean differences in the full-weighting ablation were positive, with six remaining significant after correction. Pink-noise experiments also demonstrated the improvement of soft reconstruction over hard truncation; both the pink-noise and CWRU leave-one-load-out experiments supported the performance gains of full weighting over unweighted features. Class-level analysis showed that improvements were distributed across multiple fault conditions, and the current CPU implementation requires a mean of approximately 0.31 s per window for the complete computation.

The Paderborn white- and pink-noise experiments demonstrated the benefits of continuous modal reconstruction over fixed hard truncation, while the evaluated Paderborn and CWRU experiments supported hierarchical weighting over unweighted feature fusion. These findings provide a basis for improving bearing fault diagnosis with limited training samples under the investigated conditions. Future work will extend the datasets and noise conditions and optimize computational efficiency.

## Figures and Tables

**Table 1:** Formulas and physical interpretations of the nine-dimensional time-domain statistical features.

Symbol	Feature	Formula	Interpretation and Diagnostic Role
μ	Mean	$\mu =\frac{1}{N}\sum_{i=1}^{N}x_{i}$	Describes the signal center and can reflect a DC offset.
σ	Standard deviation	$\sigma =\sqrt{\frac{1}{N}\sum_{i=1}^{N}{\left(x_{i}-\mu \right)}^{2}}$	Describes amplitude dispersion around the mean.
RMS	Root mean square	$\mathrm{RMS}=\sqrt{\frac{1}{N}\sum_{i=1}^{N}x_{i}^{2}}$	Measures the effective amplitude and overall energy level of the vibration signal.
Skew	Skewness	$\mathrm{Skew}=\frac{1}{N}\sum_{i=1}^{N}{\left(\frac{x_{i}-\mu }{\sigma }\right)}^{3}$	Characterizes asymmetry in the amplitude distribution.
Kurt	Kurtosis	$\mathrm{Kurt}=\frac{1}{N}\sum_{i=1}^{N}{\left(\frac{x_{i}-\mu }{\sigma }\right)}^{4}$	Is sensitive to localized impacts and can characterize impulsive faults.
CF	Crest factor	$\mathrm{CF}=\frac{x_{p}}{\mathrm{RMS}}$	Compares the peak with the effective amplitude, reflecting the prominence of transient impacts.
SF	Shape factor	$\mathrm{SF}=\frac{\mathrm{RMS}}{{\bar{x}}_{\mathrm{abs}}}$	Describes changes in waveform shape relative to the mean amplitude.
IF	Impulse factor	$\mathrm{IF}=\frac{x_{p}}{{\bar{x}}_{\mathrm{abs}}}$	Measures the peak relative to the mean absolute value.
CLF	Clearance factor	$\mathrm{CLF}=\frac{x_{p}}{x_{r}}$	Highlights spikes and early impacts against a low-amplitude background.

**Table 2:** Formulas and physical interpretations of the six-dimensional frequency-domain statistical features.

Symbol	Feature	Formula	Interpretation and Diagnostic Role
$\bar{A}$	Mean spectral amplitude	$\bar{A}=\frac{1}{M}\sum_{k=1}^{M}A_{k}$	Describes the average spectral amplitude level over the positive-frequency range.
$\sigma_{A}$	Standard deviation of spectral amplitude	$\sigma_{A}=\sqrt{\frac{1}{M}\sum_{k=1}^{M}{\left(A_{k}-\bar{A}\right)}^{2}}$	Measures the dispersion and variation in spectral amplitudes.
$f_{c}$	Spectral centroid	$f_{c}=\frac{\sum_{k=1}^{M}f_{k}A_{k}}{\sum_{k=1}^{M}A_{k}}$	Characterizes the center of the spectral energy distribution.
$E_{f}$	Spectral energy	$E_{f}=\sum_{k=1}^{M}A_{k}^{2}$	Describes the overall energy level in the frequency domain.
$f_{\mathrm{MS}}$	Mean square frequency	$f_{\mathrm{MS}}=\frac{\sum_{k=1}^{M}f_{k}^{2}A_{k}}{\sum_{k=1}^{M}A_{k}}$	Emphasizes the contribution of high-frequency components to the spectral distribution.
$\sigma_{f}$	Frequency standard deviation	$\sigma_{f}=\sqrt{\frac{\sum_{k=1}^{M}{\left(f_{k}-f_{c}\right)}^{2}A_{k}}{\sum_{k=1}^{M}A_{k}}}$	Describes the spectral spread around the centroid.

**Table 3:** Classes, damage types, and window counts in the main Paderborn experiments.

Label	Code	Condition Description	Windows
0	K001	Healthy bearing	200
1	KA01	Artificially machined outer-race damage	200
2	KB23	Combined inner- and outer-race damage caused by real fatigue, dominated by inner-race damage	200
3	KI01	Artificially machined inner-race damage	200

**Table 4:** Fixed protocols for the two main branches and supplementary validation. Comparisons within each row share the classifier and data splits.

Experiment	Features and Comparisons	*C*	γ	White-Noise SNR
Paderborn reconstruction	TF15; Raw/All/Hard/Soft	1	1/15	−5,−10
Paderborn weighting	Soft TF35; U/S/D/SD	10	1/35	−10
CWRU reconstruction	TF15; Raw/All/Hard/Soft	1, 10	1/150	−5,−10
CWRU weighting	Soft TF35; U/S/D/SD	10	1/35	−10

**Table 5:** Complete reconstruction accuracy under two Paderborn white-noise conditions (mean ± population standard deviation, %). All classifiers use C=1 and γ=1/15.

SNR/dB	Method	SS10	SS20	SS30
−5	Raw	74.66 ± 3.90	78.78 ± 2.84	81.30 ± 1.90
−5	VMD-All	75.08 ± 3.31	79.18 ± 2.58	81.85 ± 1.82
−5	Hard-VMD	81.05 ± 3.90	84.56 ± 2.45	85.67 ± 2.02
−5	Soft-VMD	82.24 ± 3.19	86.50 ± 2.33	88.06 ± 1.93
−10	Raw	55.33 ± 2.90	59.23 ± 2.69	61.30 ± 2.01
−10	VMD-All	56.21 ± 3.15	60.10 ± 2.58	61.81 ± 2.25
−10	Hard-VMD	60.81 ± 4.16	65.05 ± 2.81	68.19 ± 1.96
−10	Soft-VMD	64.15 ± 3.65	68.28 ± 2.22	69.95 ± 2.57

**Table 6:** Six exploratory Soft–Hard statistics. Differences and conditional marginal 95% intervals are in percentage points.

SNR/dB	Scenario	Δ	Conditional Interval	$d_{z}$	B/E/W	Holm *p*
−5	SS10	+1.1875	[0.639, 1.739]	0.786	14/2/4	0.00668828
−5	SS20	+1.9375	[1.295, 2.575]	1.462	19/1/0	0.000636696
−5	SS30	+2.3875	[1.743, 3.065]	1.780	19/1/0	0.000636696
−10	SS10	+3.3375	[2.367, 4.357]	1.855	20/0/0	0.000527693
−10	SS20	+3.2250	[2.189, 4.233]	1.238	17/1/2	0.00118633
−10	SS30	+1.7625	[0.771, 2.779]	0.857	17/0/3	0.00668828

**Table 7:** Current TF35/C10 four-method ablation (%). Accuracy is reported as mean ± population standard deviation, and macro-averaged F1 as the mean over twenty runs.

Method	Acc_10_	Acc_20_	Acc_30_	F1_10_	F1_20_	F1_30_
U	64.97 ± 4.23	69.19 ± 3.45	71.03 ± 2.98	64.88	68.88	70.79
S	65.92 ± 4.02	70.30 ± 2.97	72.79 ± 2.59	65.75	69.95	72.52
D	65.72 ± 4.25	69.88 ± 3.28	72.10 ± 2.66	65.63	69.53	71.81
SD	66.53 ± 3.62	71.15 ± 2.90	73.26 ± 2.49	66.32	70.75	72.96

**Table 8:** Nine exploratory comparisons of the full method against three ablations. Differences and conditional intervals are in percentage points; significance is based on Holm correction within the family of nine comparisons.

Scenario	Comparison	Δ	Conditional Marginal 95% Interval	Holm *p*	Significant
SS10	SD–U	+1.5500	[0.880, 2.292]	0.0267078	Yes
SS10	SD–S	+0.6000	[0.231, 0.991]	0.220824	No
SS10	SD–D	+0.8000	[0.198, 1.443]	0.220824	No
SS20	SD–U	+1.9625	[1.268, 2.613]	0.00529709	Yes
SS20	SD–S	+0.8500	[0.426, 1.257]	0.0354158	Yes
SS20	SD–D	+1.2750	[0.688, 1.841]	0.0123482	Yes
SS30	SD–U	+2.2375	[1.535, 2.920]	0.00529709	Yes
SS30	SD–S	+0.4750	[0.101, 0.850]	0.220824	No
SS30	SD–D	+1.1625	[0.598, 1.728]	0.0354158	Yes

**Table 9:** Comparison of entropy representations using the same Soft-VMD, TF15, and fixed C10 classifier (%). Accuracy is reported as mean ± population standard deviation, and F1 as the mean over twenty runs.

Entropy/Fusion	Acc_10_	Acc_20_	Acc_30_	F1_10_	F1_20_	F1_30_
MFE	61.43 ± 3.26	65.40 ± 2.20	66.95 ± 1.99	61.38	65.09	66.62
CMFE	61.25 ± 3.75	66.36 ± 3.36	68.89 ± 2.81	61.04	65.95	68.41
RCMFE	61.29 ± 3.70	66.25 ± 3.44	68.71 ± 2.95	61.07	65.84	68.21
RCMFDE-U	64.97 ± 4.23	69.19 ± 3.45	71.03 ± 2.98	64.88	68.88	70.79
SD	66.53 ± 3.62	71.15 ± 2.90	73.26 ± 2.49	66.32	70.75	72.96

**Table 10:** Complete mean accuracy under pink noise at −10 dB (%). The three noise realizations are trained and evaluated separately and then averaged with equal weights.

Branch	Method	SS10	SS20	SS30
TF15/C1	Raw	58.82	63.39	66.01
TF15/C1	VMD-All	58.86	62.83	65.45
TF15/C1	Hard-VMD	46.59	50.85	53.63
TF15/C1	Soft-VMD	52.59	56.84	59.17
TF35/C10	U	48.64	53.60	57.46
TF35/C10	S	49.28	54.31	58.05
TF35/C10	D	51.69	57.54	61.29
TF35/C10	SD	52.17	57.97	61.51

**Table 11:** Equal-weight averages of the means for the four CWRU held-out loads (%). Each load mean is based on twenty training-sample draws.

Method	Acc_10_	Acc_20_	Acc_30_	F1_10_	F1_20_	F1_30_
U	81.26	87.94	91.11	81.28	87.91	91.05
S	83.33	90.25	93.01	83.35	90.22	92.94
D	88.98	95.86	97.83	88.96	95.86	97.83
SD	89.13	95.57	97.79	89.11	95.57	97.79

**Table 12:** Directly measured time from a single window to a predicted label. Each row includes eight windows with three repetitions per window, excluding acquisition, noise generation, and I/O.

Branch	SNR/dB	Mean	Median	Unit
TF15/C1	−5	297.9394	308.5103	ms/window
TF15/C1	−10	303.3888	272.4479	ms/window
TF35/C10	−10	308.5027	278.0143	ms/window

## Data Availability

The original data are publicly available from the Paderborn University KAt-DataCenter (https://mb.uni-paderborn.de/en/kat/research/bearing-datacenter, accessed on 25 August 2026) and the CWRU Bearing Data Center (https://engineering.case.edu/bearingdatacenter/download-data-file, accessed on 25 August 2026).

## Appendix A. Complete Supplementary Results

### Appendix A.1. Reconstruction Macro-Averaged F1

**Table A1:** Complete macro-averaged F1 for the first branch (mean ± population standard deviation, %).

SNR/dB	Method	SS10	SS20	SS30
−5	Raw	73.66 ± 4.14	77.90 ± 2.83	80.53 ± 1.97
−5	VMD-All	74.04 ± 3.47	78.28 ± 2.52	81.09 ± 1.82
−5	Hard-VMD	80.24 ± 3.78	83.95 ± 2.47	85.15 ± 2.09
−5	Soft-VMD	81.43 ± 3.16	85.95 ± 2.44	87.65 ± 2.03
−10	Raw	53.93 ± 3.11	57.98 ± 2.78	60.01 ± 2.12
−10	VMD-All	54.65 ± 3.30	58.68 ± 2.77	60.32 ± 2.38
−10	Hard-VMD	59.33 ± 3.79	63.61 ± 2.99	66.64 ± 2.09
−10	Soft-VMD	62.58 ± 3.64	67.04 ± 2.32	68.60 ± 2.71

### Appendix A.2. Complete Parameter Diagnostics on a Fixed Training Subset

**Table A2:** One-factor-at-a-time local diagnostics on the fixed outer training set of the first SS30 run (%).

Factor	Value/Comparator	Acc_−5_	Acc_−10_	F1_−5_	F1_−10_
Comparator	Raw	84.17	67.50	83.78	66.75
Comparator	Hard-VMD	87.50	65.00	87.15	64.23
Comparator	VMD-All	85.00	64.17	84.56	63.27
Comparator	Soft-VMD	90.83	69.17	90.60	68.93
λ	0	87.50	65.00	87.15	64.23
λ	0.05	89.17	64.17	88.80	63.47
λ	0.1	90.00	67.50	89.70	67.03
λ	0.15	90.83	69.17	90.60	68.93
λ	0.2	90.83	66.67	90.60	66.41
λ	0.3	90.83	67.50	90.60	66.81
λ	0.5	87.50	70.00	87.19	69.50
λ	0.8	85.83	67.50	85.69	66.84
λ	1	85.00	64.17	84.56	63.27
Retained prefix	2	90.00	70.83	89.74	68.76
Retained prefix	3	90.83	69.17	90.60	68.93
Retained prefix	4	90.00	63.33	89.80	62.83
*K*	6	82.50	60.00	82.42	58.98
*K*	7	90.83	69.17	90.60	68.93
*K*	8	90.00	74.17	89.72	73.75
α	100	87.50	66.67	87.25	66.26
α	200	90.83	69.17	90.60	68.93
α	400	90.00	69.17	89.72	68.83

### Appendix A.3. Complete Figures for Pink Noise and CWRU

**Table A3:** Complete mean accuracy of the CWRU reconstruction branch (%), aggregated with equal weights across the four held-out loads.

C	SNR/dB	Method	SS10	SS20	SS30
1	−5	Raw	86.24	90.96	94.19
1	−5	VMD-All	88.09	92.30	94.64
1	−5	Hard-VMD	85.58	92.13	93.97
1	−5	Soft-VMD	90.87	96.81	97.75
1	−10	Raw	81.82	89.32	93.86
1	−10	VMD-All	81.98	89.19	92.80
1	−10	Hard-VMD	73.83	80.87	84.46
1	−10	Soft-VMD	77.23	85.67	88.53
10	−5	Raw	97.89	99.02	99.18
10	−5	VMD-All	97.61	98.81	98.93
10	−5	Hard-VMD	96.30	98.64	98.94
10	−5	Soft-VMD	98.00	99.19	99.40
10	−10	Raw	96.08	98.30	98.88
10	−10	VMD-All	95.43	97.90	98.49
10	−10	Hard-VMD	89.20	96.26	98.23
10	−10	Soft-VMD	90.01	95.17	97.25
